# Urban–rural disparity of social vulnerability to natural hazards in Australia

**DOI:** 10.1038/s41598-022-17878-6

**Published:** 2022-08-11

**Authors:** Siqin Wang, Mengxi Zhang, Xiao Huang, Tao Hu, Qian Chayn Sun, Jonathan Corcoran, Yan Liu

**Affiliations:** 1 School of Earth and Environmental Sciences, University of Queensland, Brisbane, QLD 4067 Australia; 2 Department of Nutrition and Health Science, Ball State University, Indiana, USA; 3 Department of Geosciences, University of Arkansas, Fayetteville, Arkansas USA; 4 Department of Geography, Oklahoma State University, Oklahoma, USA; 5 School of Science, Geospatial Sciences, RMIT University, 124 La Trobe Street, Melbourne, VIC 3000 Australia

**Keywords:** Climate-change adaptation, Climate-change impacts, Climate-change mitigation, Environmental impact, Sustainability

## Abstract

Assessing vulnerability to natural hazards is at the heart of hazard risk reduction. However, many countries such as Australia lack measuring systems to quantity vulnerability for hazard risk evaluation. Drawing on 41 indicators from multiple data sources at the finest spatial unit of the Australian census, we re-forged the Cutter’s classic vulnerability measuring framework by involving the ‘4D’ quantification of built environment (diversity, design, density and distance), and constructed the first nationwide fine-grained measures of vulnerability for urban and rural locales, respectively. Our measures of vulnerability include five themes—(1) socioeconomic status; (2) demographics and disability; (3) minority and languages; (4) housing characteristics; and (5) built environment—that were further used to assess the inequality of vulnerability to three widely affected natural hazards in Australia (wildfires, floods, and earthquakes). We found the inequality of vulnerability in the affected areas of the three hazards in eight capital cities are more significant than that of their rural counterparts. The most vulnerable areas in capital cities were peri-urban locales which must be prioritised for hazard adaptation. Our findings contribute to the risk profiling and sustainable urban–rural development in Australia, and the broad understanding of place-based risk reduction in South Hemisphere.

## Introduction

It is projected that more than two-thirds of the world population will live in urban areas by 2050^1^. The increasing urban–rural disparity in terms of socioeconomic status, access of social resources, and built environment poses emerging challenges for natural hazard risk management^2^. Assessing vulnerability and identifying the locations of vulnerable populations lie at the heart of hazard risk reduction, though it faces enormous challenges that are distinct within and across urban and rural environments^3^. A natural hazard occurring in urban areas may induce more severe loss of life and assets compared to rural areas, due to the high density of population and infrastructure. While hazard-affected areas and victims in urban areas may receive more efficient and prompt responses, help, and delivery of living or medical resources than those in rural areas, due to the location of cities that can be easily accessed^4,5^. Social groups with different demographic characteristics (e.g., the elderly and children) and socioeconomic status (e.g., low-income and unemployed) have different capacities to adapt to natural hazards^6,7^. The disproportional distributions of social groups across urban and rural spaces further widen the urban–rural disparity of natural hazard risk management^8^. A system of measuring the vulnerability of urban and rural areas to natural hazard risk is important in its capacity to guide place-based risk profiling and recovery.

However, measuring vulnerability to natural hazard risk is challenging due to the complexity of the concept. Vulnerability to natural hazard risks has been defined by considering two viewpoints^9^. The first perceives vulnerability as the system of being physically exposed to a hazard^10^. Exposure is usually measured by the number or density of people and buildings in hazard-affected areas^11^. The second viewpoint considers vulnerability as a more complex capacity of society and individuals to cope with hazard and damage^12,13^. In this case, vulnerability often refers to social vulnerability that was quantified in the classic work by Cutter et al. proposed a factor analytic framework to construct the social vulnerability index of U.S. counties^6^. This framework has been applied to many countries including Norway^14^, Nepal^15,16^, China^17^, Bangladesh^18^, Portugal^19^, India^20,21^, Brazil^22^, Colombia^23^, and Zimbabwe^24^. A wide range of factors used to measure vulnerability include demographic and socioeconomic status^14,15,25^, housing^6,13,26–29^, development of facilities^7,20,27,30^, and medical services^14,17,20,27,30^. Those factors reflect social inequalities, shaping the susceptibility of various groups to hazards and governing their capacity to respond^6^. Although the Cutter’s framework for measuring vulnerability also includes some built environment indicators, such as the density of commercial and industrial development^26,31^, there is arguably an opportunity to extend this to include additional dimensions (i.e., density, diversity, design, and distance) to better reflect place inequalities in face of natural hazards^32^. In addition, such measures of vulnerability that have been employed in the aforementioned countries have not yet been grounded in Oceanian countries including Australia—a nation that needs further efforts to enhance its resilience to climate change.

Australia has a long history of natural hazards including wildfires, floods, earthquakes, storms, cyclones, and landslides. The impact associated with hazards varies and can range from frequent moderate impacts (e.g., wildfires) to rare but potentially catastrophic impacts (e.g., earthquakes). The frequency and severity of such hazards are expected to increase in the future due to global climate change^33,34^, but the Australian government lacks of action to cope with such impacts^35^. Australia adopted the New Urban Agenda at Habitat III in 2016 and became a party to the Paris Climate Agreement in the same year^36^. What this means specifically for hazard risk assessment in Australia has not yet been developed to any significant degree. While various strategies and government reports exist in Australia to profile Australia’s vulnerability (e.g., Department of Home Affairs, National Resilience Taskforce, CSIRO, Australia Geoscience), these are largely qualitative evaluation of vulnerability based on data from interviews of experts and public workshop. The limited review work^37,38^ and quantitative studies^39–42^ measuring vulnerability were undertaken only for specific cities (e.g., Sydney, Gold Coast, Wollongong) or state (e.g., New South Wales), with data outdated at the time of publication. The Australian Bureau of Statistics developed the Socio-Economic Indexes for Areas (SEIFA), which contains the indices of advantage, disadvantage, economic resources, education, and occupation to reflect the social and welfare policy development in Australia^43^. However, SEIFA mainly captures the demographic and socioeconomic profiles of the Australian population; it lacks the capability to capture the vulnerability from the housing and built environment dimensions as in the Cutter framework^6^.

**Figure 1:**
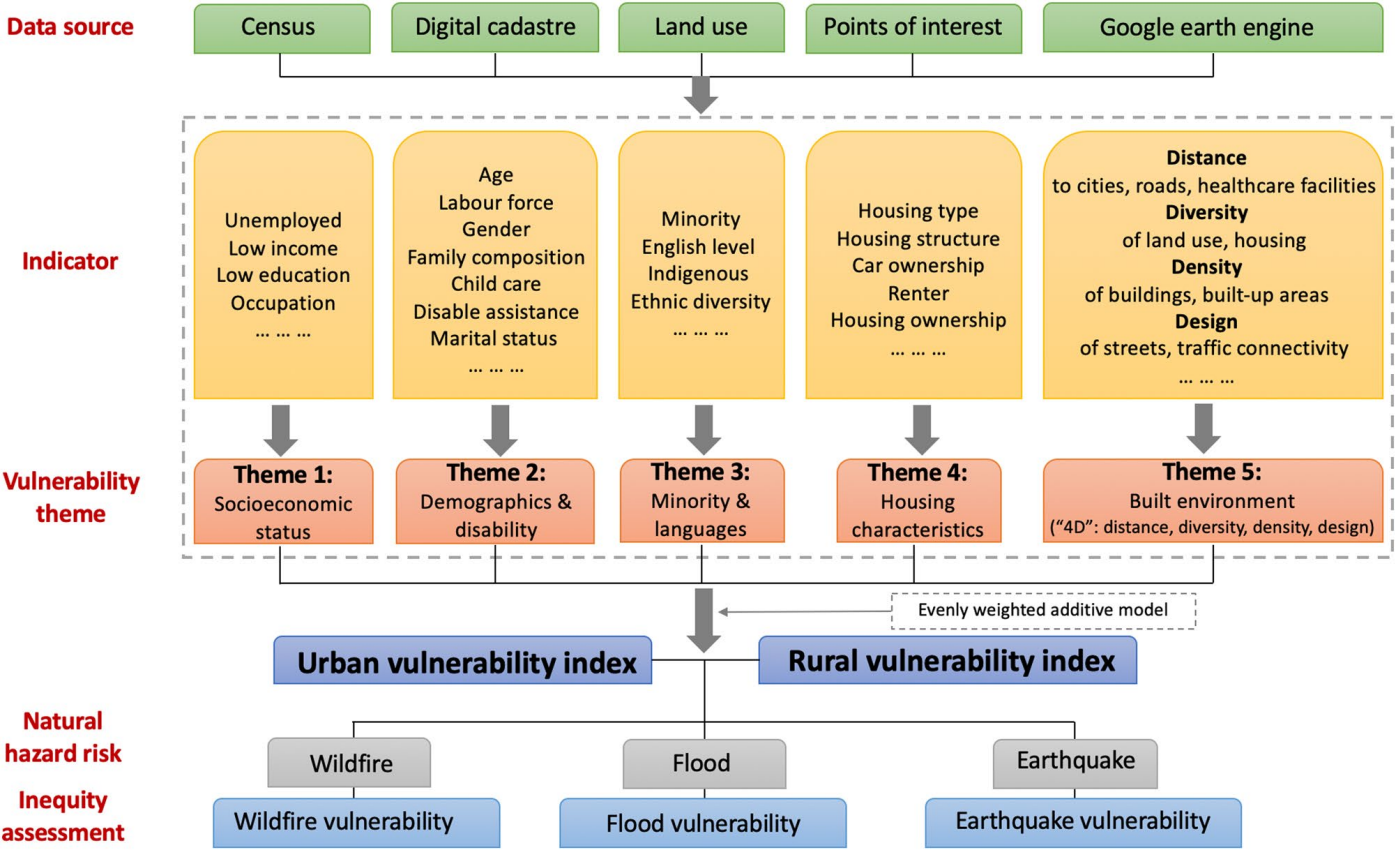
Research design and analytical framework. Data retrieval from the five sources and data processing are detailed in Methods. The definition and measure of each indicator with data sources are provided in Supplementary Table S2.

To address this knowledge deficit, this study aims to construct the first Australian nationwide fine-grained measures of vulnerability for urban and rural space, respectively, and evaluate the inequality of vulnerability to three widely affected natural hazards in Australia. We select three types of natural hazards—wildfires, floods, and earthquakes—as wildfires and floods are the two most notable and common natural hazards in Australia, and earthquakes have a wider impact on both inland and coastal regions compared to storm surges and cyclones that predominantly affect coastal regions. We follow the analytical framework (Fig. 1) and first draw on multiple data sources to retrieve 41 indicators at the smallest census unit of Australia (Statistical Areas level 1). The rationale of selecting these 41 indicators (including concepts, metrics, and hypothesised effect on vulnerability) is detailed in Supplementary Table 1. Methodologically, we re-forge the Cutter framework by additionally involving the ‘4D’ quantification of built environment, and established two measuring systems of vulnerability for urban and rural areas, respectively, to the analytical framework (Fig. 1). We further classify our measures of vulnerability in five themes—Theme 1 (socioeconomic status), Theme 2 (demographics and disability), Theme 3 (minority and languages), Theme 4 (housing characteristics) which were used by US Centres of Disease Control and Prevention (CDC) in their measures of vulnerability, plus the additional Theme 5 of built environment which has been include in the Cutter’s work^6^. Such classifications avoid the complex and evolving definitions of exposure, sensitivity, and vulnerability which were somehow overlapping or controversial to some degree in early studies. We further examine the inequality of vulnerability in the hazard-affected areas across eight capital cities and their rural counterparts (Supplementary Note 1 and Fig. S1). Our results contribute to profiling Australia’s vulnerability, and provide quantitative evidence for place-based risk reduction and the sustainability of urban–rural development.

## Results

### Measures of the vulnerability index

**Figure 2:**
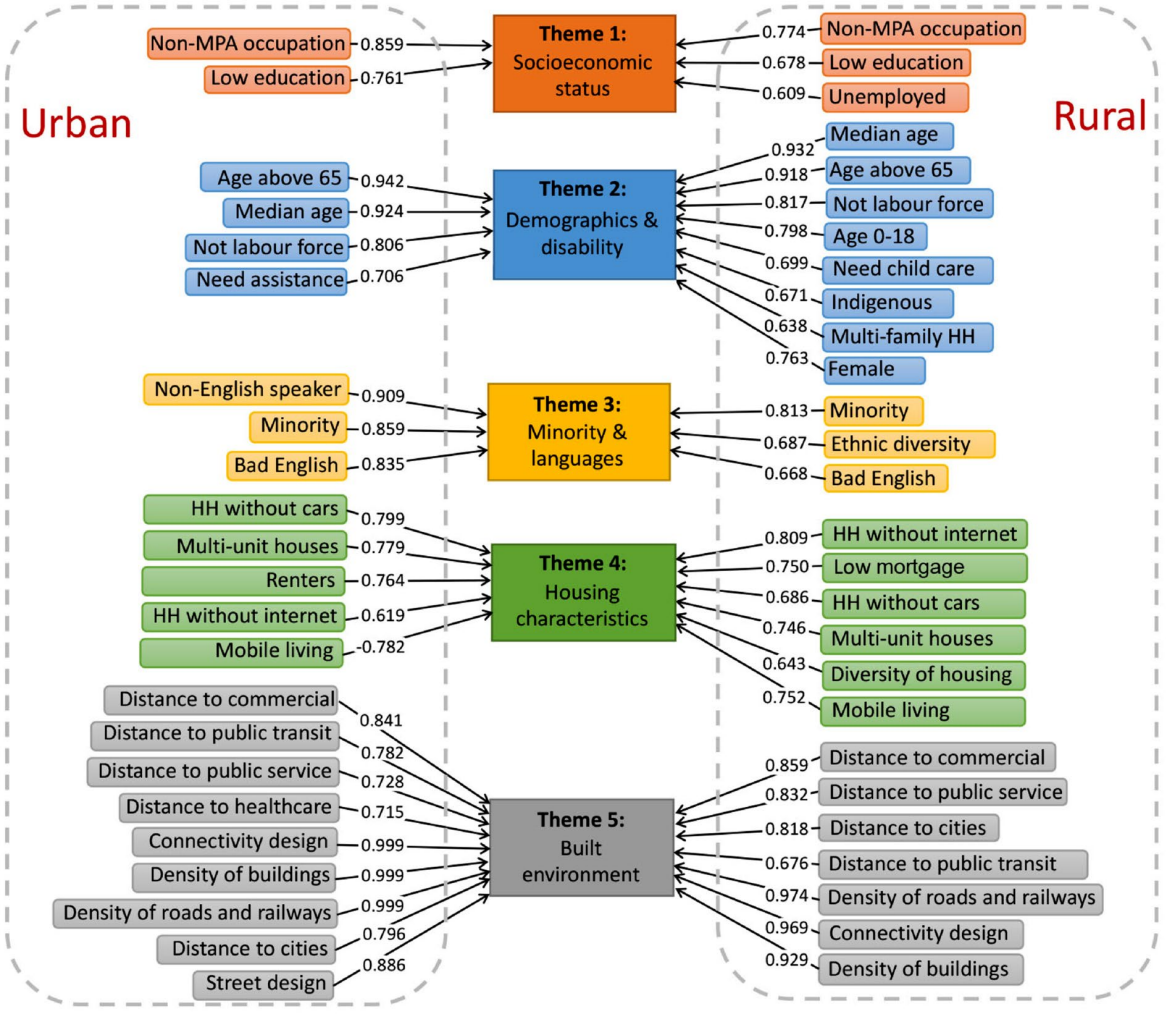
Reclassification of indicators in five themes for urban and rural space. MPA: managers, professionals, and administrators; HH: households; the numbers on the top of the arrows denote the loading factors of each indicator (Supplementary Tables S3 and S4), reflecting how much each indicator contributes to each principal component that is further classified to a particular theme. In urban areas, there are 10 principal components identified by the principal component analysis based on 23 indicators to measure the vulnerability index (Supplementary Table S5); they explain a total of 69.45% of data variance across SA1 areas. In rural areas, there are 11 principal components identified by the principal component analysis based on 27 indicators to measure the vulnerability index (Supplementary Table S6); they explain a total of 68.52% of data variance across SA1 areas. Principal components were further classified to five themes.

We generated an overall vulnerability index by taking into account indicators under all five themes, and one individual vulnerability index under each theme. Most of the indicators in Themes 1–5 for urban and rural areas have positive loading factors ($\alpha$), reflecting the positive relationship between the indicators and vulnerability (Fig. 2). Specifically, in Theme 1 of socioeconomic status, non-MPA (managers, professionals and administrators) occupation ($\alpha$ = 0.859) and low education level ($\alpha$ = 0.761) are identified as indicators significantly contributing to the vulnerability in urban areas. It means that urban areas with higher proportions of people working in non-MPA occupation and with low education level tend to be more vulnerable (a higher level of vulnerability)—we also observed this finding in the literature^7,14,20,28,44–46^. These indicators explain 3.12% of the variation among urban areas (Supplementary Table S3). On the other hand, the factor unemployed ($\alpha$ = 0.609) was identified to contribute to the vulnerability in rural areas, indicating that rural areas with high employment rate are more vulnerable with greater impacts from natural hazards; these areas are also typically slower in their recovery from disasters. The interpretation of identified indicators for Themes 2–5 is detailed in Supplementary Note 2.

### Urban–rural disparity of vulnerability

**Figure 3:**
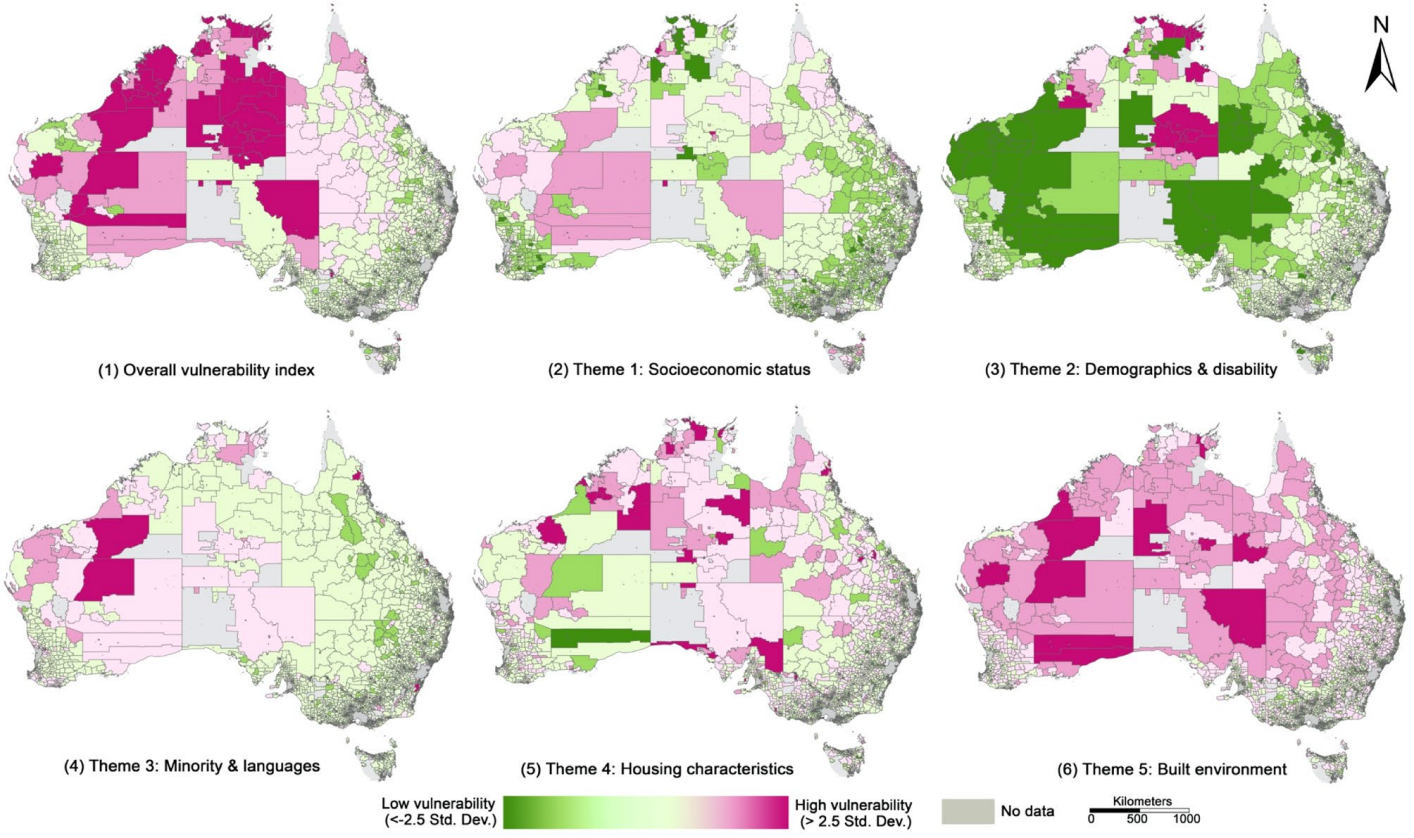
Spatial patterns of the vulnerability index in rural areas for (1) overall (combining five themes); (2) Theme 1 (socioeconomic status); (3) Theme 2 (demographics and disability); (4) Theme 3 (minority and languages); (5) Theme 4 (housing characteristics); (6) Theme 5 (built environment). The vulnerability index is classified by standard deviation with a colour ramp from green indicating low vulnerability (smaller than -2.5 standard deviation) to dark pink indicating high vulnerability (larger than 2.5 standard deviation). Grey areas have no data given these areas with the number of populations less than 50 contain census data randomly assigned by Australian Bureau of Statistics due to the concern of data privacy.

**Figure 4:**
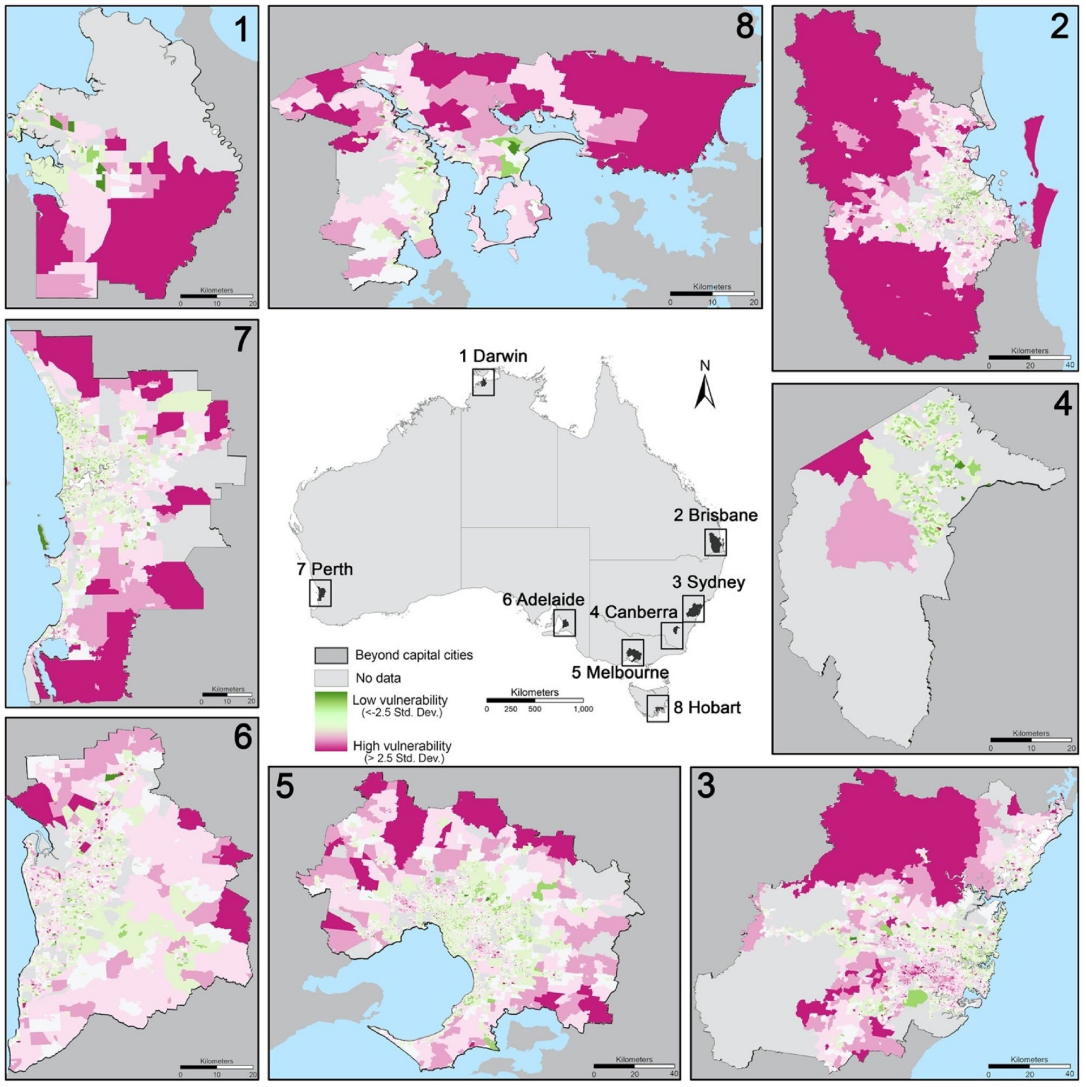
Spatial patterns of the vulnerability index in urban areas of eight capital cities: (1) Darwin (the capital city of Northern Territory); (2) Brisbane (the capital city of Queensland) ; (3) Sydney (the capital city of New South Wales); (4) Canberra (Australian Capital Territory (ACT)) ; (5) Melbourne (the capital city of Victoria); (6) Adelaide (the capital city of South Australia); (7) Perth (the capital city of West Australia); (8) Hobart (the capital city of Tasmania). The spatial patterns of the vulnerability index in each theme are displayed in Supplementary Figs. S2–S6. The identification of the most vulnerable urban areas (> 2.5 standard deviation) in different themes are presented in Supplementary Table S7. More specifically, for Theme 2 (Supplementary Fig. S3), the most vulnerable areas in terms of demographic composition and disability have less obvious patterns that can be generalised but more spread out sporadically across entire urban space outside of inner cities in Sydney, Brisbane, Adelaide, Perth, and ACT. For Theme 3 (Supplementary Fig. S4), the most vulnerable areas in terms of minority status and languages appear in Sydney (e.g., Liverpool and Blacktown), Melbourne (e.g., Springvale); in the south and southwest of Brisbane (e.g., Sunnybank, Sunnybank Hills); in Adelaide (e.g., Parafield), Perth (e.g., Jandakot) and Darwin (e.g., Humpty Doo). For Theme 4 (Supplementary Fig. S5), the most vulnerable areas in terms of housing types and transportation appear do not have specific patterns to generalise, but more sporadically appear in a few suburbs in each capital city (detailed in Supplementary Note 3). For Theme 5 (Supplementary Fig. S6), the most vulnerable areas in terms of built environment in capital cities share some common patterns—largely appearing in peri-urban locales, far away from inner cities.

**Figure 5:**
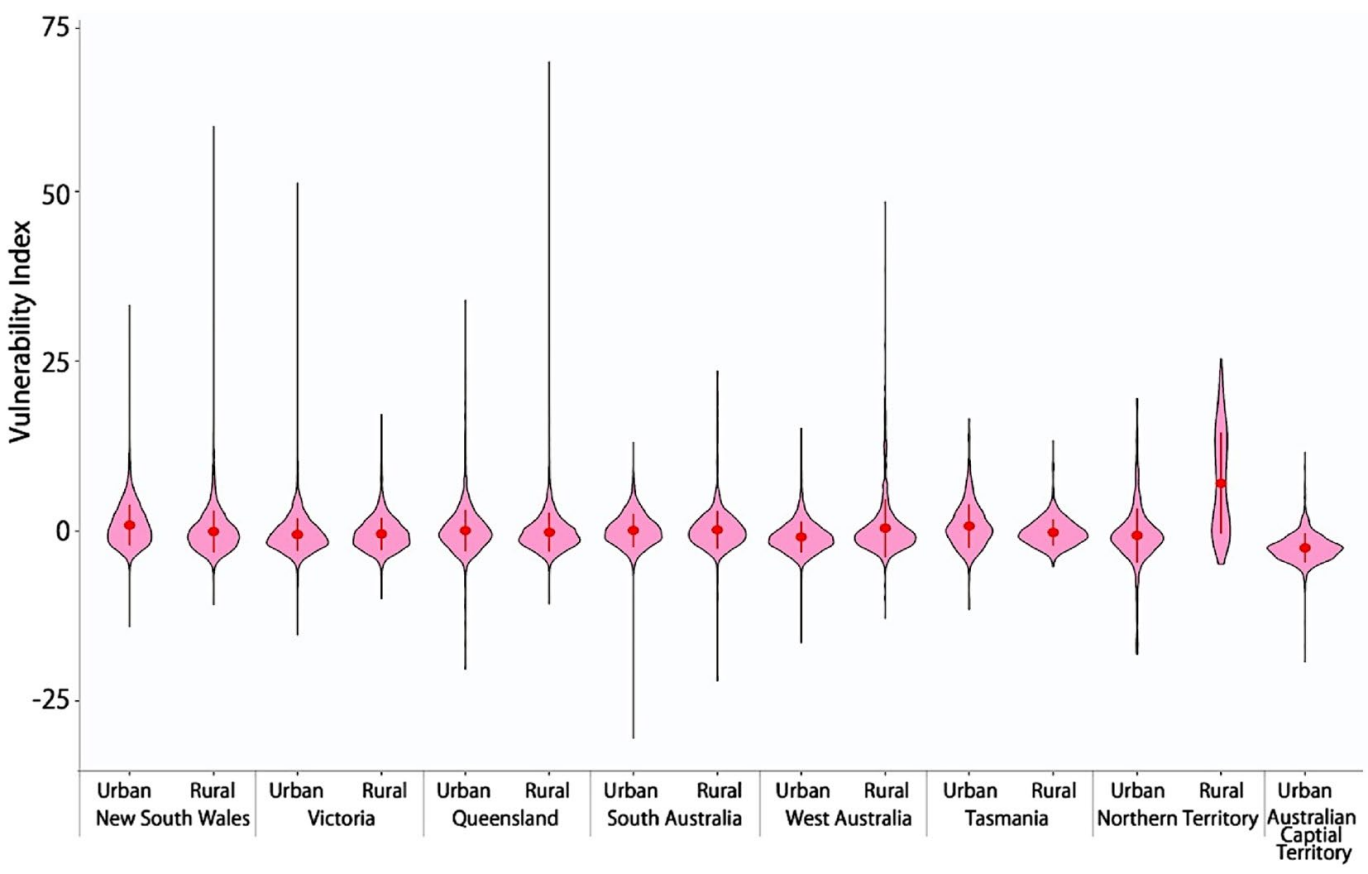
Violin plots showing the statistical distribution of the vulnerability index in urban and rural areas. Statistical details are provided in Supplementary Table S8. The vertical black line indicates the range (maximum and minimum) of the vulnerability index. The width of water-drop pink areas indicates the density distribution of the vulnerability index; a wider width means more spatial units (SA1s) concentrate in that vulnerability index. The red dot indicates the mean of the vulnerability index and the vertical red line represents 95% confidence intervals. X axis presents the ACT as urban space together with seven states which were divided into urban and rural space. The ranges of the vulnerability index across are substantially different urban and rural space. In Victoria and Tasmania, the vulnerability index in urban areas (e.g., Melbourne: − 15.260 to 51.240) have wider ranges than that in its rural areas; while in other states (e.g., New South Wales, Queensland, and West Australia), the vulnerability index in urban areas (e.g., Sydney: − 14.038 to 33.238), have wider ranges than the vulnerability index in rural areas (Supplementary Table S8).

**Figure 6:**
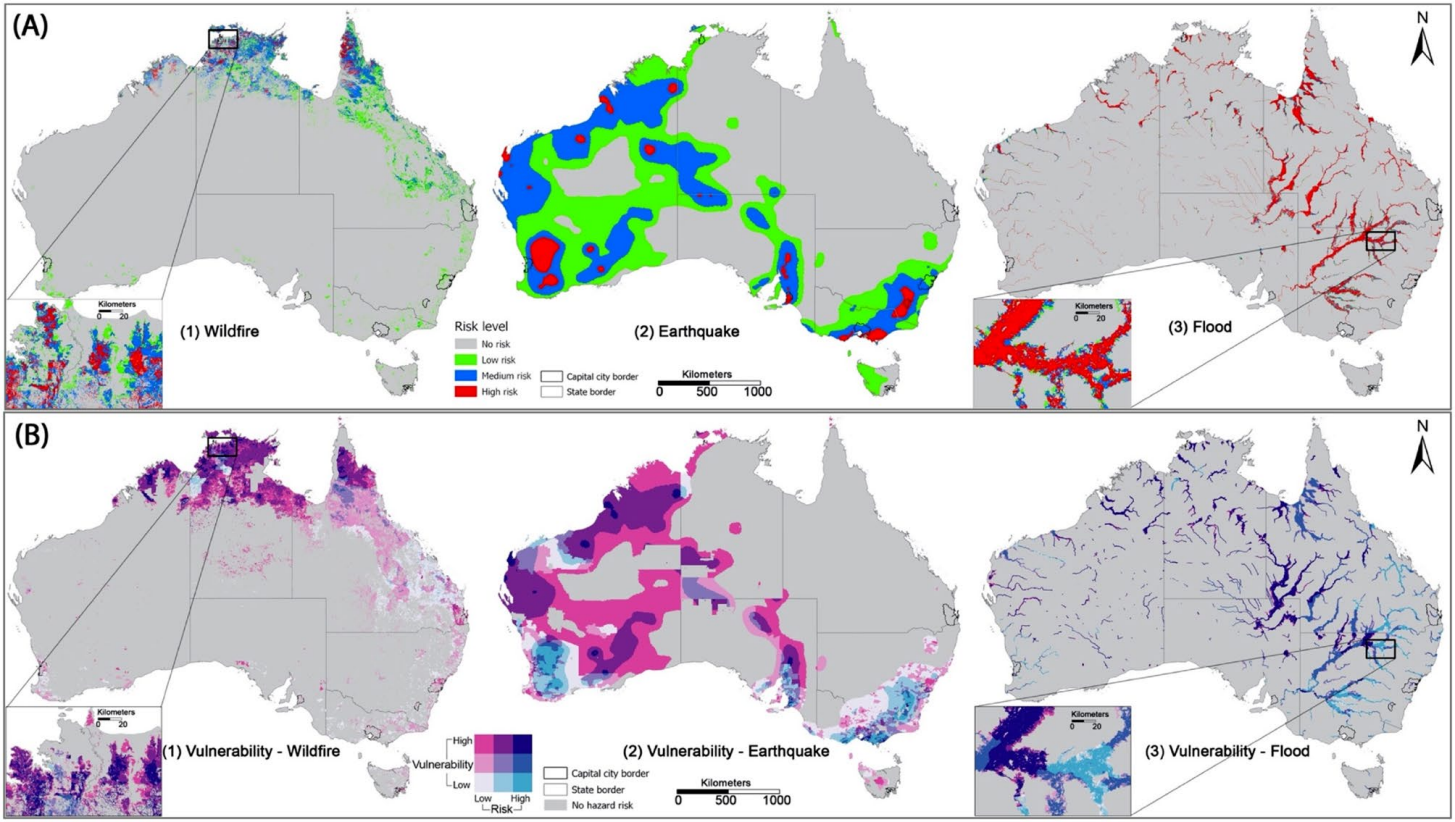
Spatial patterns of (A) three popular natural hazards in Australia and (B) the vulnerability index in hazard-affected areas. In (A), the classification of risk levels is detailed in Method. The risk of natural hazards and level of vulnerability are mapped out using a two-dimensional legend. The vertical colour ramp represents the level of vulnerability while the horizonal colour ramp represents the level of hazard risks. Our interest focuses on the most vulnerable areas with the highest level of risks (dark blue in the northeast corner of the legend). For wildfire, the highly risky and vulnerable areas appear in the north of West Australia, Northern Territory and Queensland (Supplementary Note 4 and Table S10). For earthquakes, the highly risky and vulnerable areas spread out sporadically in Exmouth and Roebuck in the northwest coast of West Australia and East Pilbara in the Midwest of West Australia, outback areas in South Australia, Wellington, Yarram, Foster, and areas along Western Port Bay in Victoria. For flood, the highly risky and vulnerable areas are more prevalent along the rivers, streams and hydrological network in the southwestern inland of Queensland and northeast corner of South Australia (Supplementary Table S10). For wildfire, the highly risky and vulnerable areas mainly appear in the north of West Australia (e.g., Derby), the north of Northern Territory (e.g., West Arnhem, Kakadu National Park, Daly River), and the north peninsula of Queensland (e.g., Cape York). The identified names of the most vulnerable areas in hazard-affected areas are presented in Supplementary Table S10.

The spatial patterns of the vulnerability index for rural and urban areas are shown in Fig. 3 and 4, respectively. The rural vulnerability index (Fig. 3) is displayed at the national scale given the rural areas occupy most of the national territory where deserts, wildness, national parks and conservation areas are largely located, and 28% of the Australian population inhabit. The names of the most vulnerable rural areas under each theme are presented in Supplementary Table S7. In general, for the overall vulnerability index (Fig. 3-1), remote areas towards inland (dark pink) are more vulnerable and areas towards coastal regions and surrounding capital cities (light green) are less vulnerable. The large areas with high vulnerability visible in Fig. 3 include the midwest of West Australia, central Northern Territory, and the northeast of South Australia (Supplementary Table S7). A similar pattern is observed for the vulnerability index under Theme 1 (Fig. 3-2) that remote areas towards inland (pink) are more vulnerable where higher proportions of population with non-MPA occupation, low education, and unemployment reside. For Theme 2 (Fig. 3-3), the central and northern parts of Norther Territory (e.g., indigenous protected areas east to Alice Springs and Barkly) and the areas around Karlamilyi National Park in the north of West Australia (dark pink in Fig. 3C) have the highest level of vulnerability, identified by a set of indicators (e.g., indigenous population shown in Fig. 2). For Theme 3 (Fig. 3-4), the most vulnerable areas are observed in Meekatharra and East Pilbara in the midwest of West Australia. For Theme 4 (Fig. 3-5), the distributions of the most vulnerable areas are dispersed, sporadically spreading out in West Australia, Northern Territory, and South Australia (Supplementary Table S7). Finally, for Theme 5 (Fig. 3-6), most of the regional and rural areas located in the inland are more vulnerable than areas closer to the coastal regions (Supplementary Table S7).

The spatial distributions of the vulnerability index in eight capital cities show some common patterns (Fig. 4)—peri-urban areas tend to be more vulnerable than inner-city areas and middle-ring urban areas. When it breaks down to each theme (Supplementary Figs. S2–S6), the spatial patterns of the vulnerability index are substantially different across themes. The names of the most vulnerable areas in each theme in each city are presented in Supplementary Table S7 and detailed in Supplementary Note 3. In general, for Theme 1 (Supplementary Fig. S2), the most vulnerable areas in terms of socioeconomic status appear in the west, southwest, and the south of Sydney (Supplementary Note 3); in the north and southeast Melbourne close to the inner city (e.g., Clayton); in the south and southwest of Brisbane (e.g., Logan, Beenleigh, Ipswich); and a number of suburbs in Adelaide, Perth, Darwin and Hobart. The spatial patterns of vulnerability in Theme 2–5 are provided in Supplementary Figs. S3–S6. The identified names of the most vulnerable urban areas (> 2.5 standard deviation) in different themes are presented in Supplementary Table S7.

The violin plots compare the statistical distributions of the vulnerability index in urban and rural areas (Fig. 5). Except for the rural space in Northern Territory with the mean value of the vulnerability index as 7.041, the distributions of the vulnerability index (pink areas in Fig. 5) in most of states across urban and rural space are largely similar with the mean values around 0 (from − 2.446 in ACT to 0.897 in Sydney, urban areas in New South Wales; Supplementary Table S8). However, the ranges of the vulnerability index are substantially different across the urban and rural space (Fig. 5 caption). We also observe that the inequality index in urban areas is larger than that in rural areas across seven states (Supplementary Fig. S7 and Table S9) in each setting of $k$ values. It means that vulnerability in urban areas (capital cities) tends to be less equally distributed compared to rural areas.

### Inequality of vulnerability in hazard-affected areas

**Figure 7:**
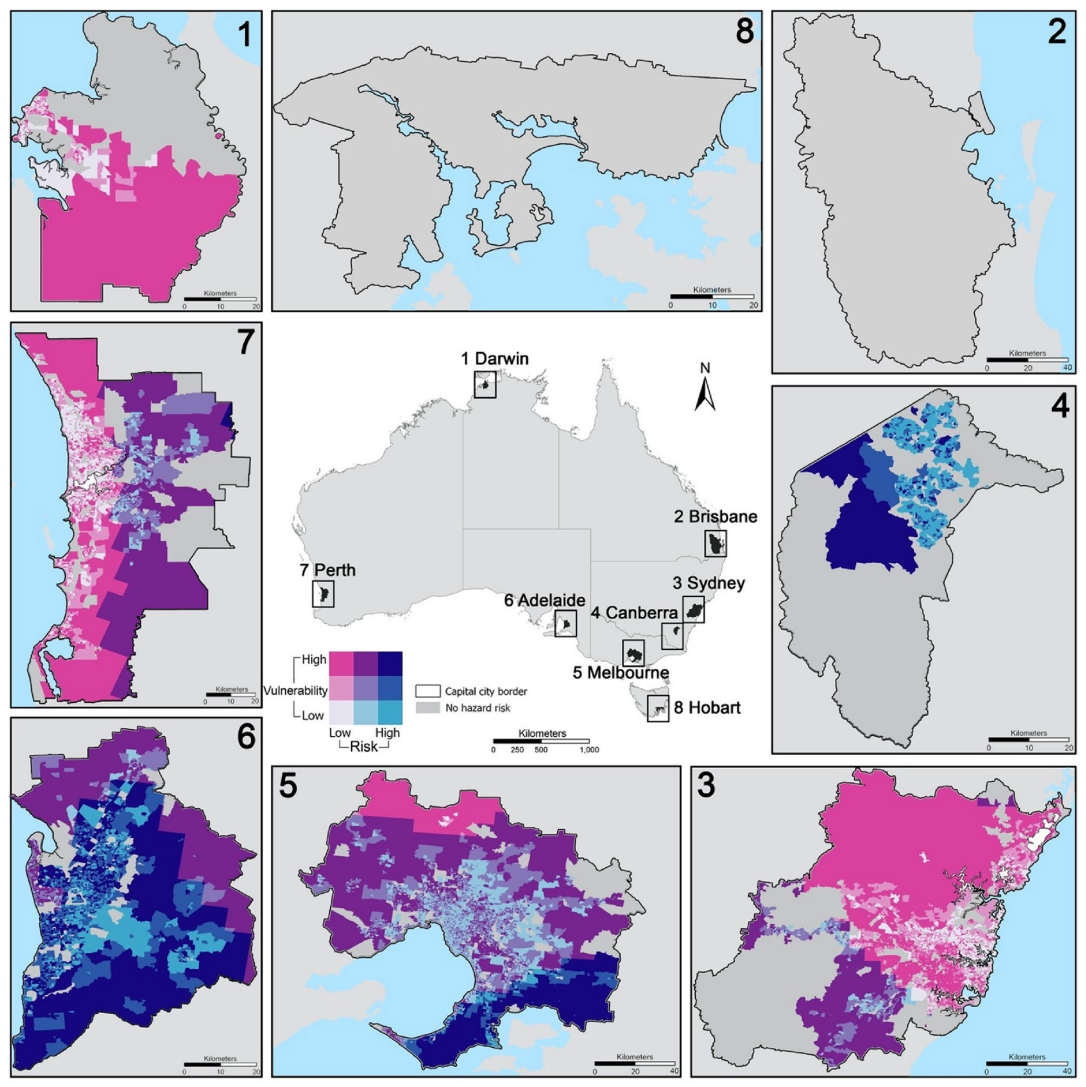
The enlarged capital city map showing the most vulnerable areas highly risky in earthquakes. Hobart and Brisbane have no earthquake-affected areas. The most vulnerable areas highly risky in earthquakes (dark blue areas) appear in the southwest of ACT, the south coastal areas of Melbourne along the Western Port Bay as well as occupy the major areas of Adelaide; while the highly vulnerable areas in the east of Perth, the south of Sydney and the south of Darwin though they are not subject to high levels of earthquake risks. In addition, there are no earthquake-affected areas Hobart and Brisbane. The spatial patterns of most vulnerable areas highly risky in wildfire and flood are displayed in Supplementary Figs. S8 and S9; the names of these vulnerable areas are detailed in Supplementary Note 5 and Table S10.

**Figure 8:**
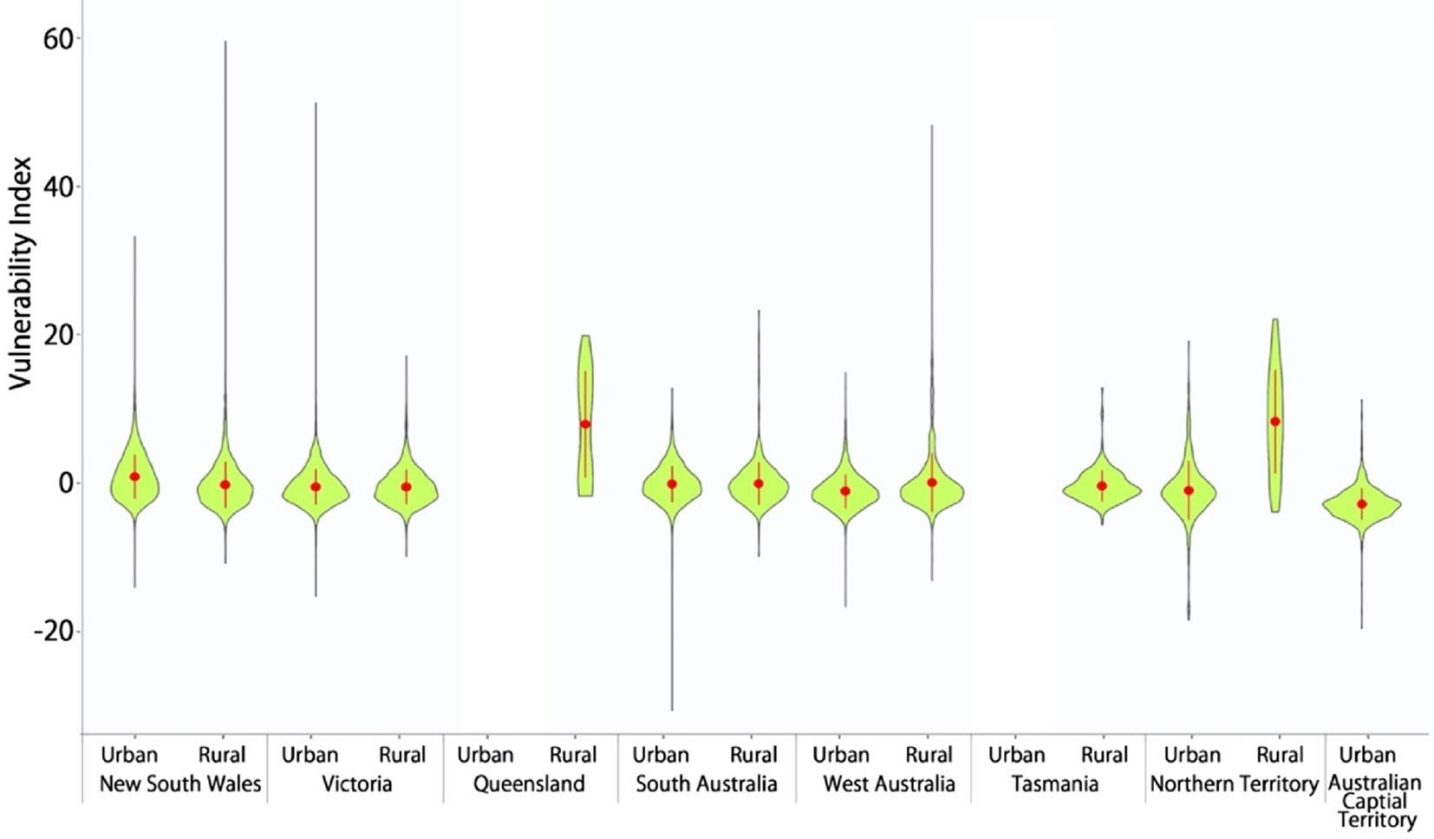
Violin plots showing the statistical distribution of the vulnerability index in earthquake-affected areas. Statistical details are provided in Supplementary Table S11. The vertical black line indicates the range (maximum and minimum) of the vulnerability index. The width of water-drop pink areas indicates the density distribution of the vulnerability index; a wider width means more spatial units (SA1s) concentrate in that vulnerability index. The red dot indicates the mean of the vulnerability index and the vertical red line represents 95% confidence intervals. X axis presents the ACT as urban space together with seven states which were divided into urban and rural space. In New South Wales and West Australia, the range of the vulnerability index in earthquake-affected rural areas is much wider than that in urban areas, while oppositely in Victoria, South Australia and Northern Territory. In particular, the vulnerability index in earthquake-affected areas ranges low in Adelaide (− 30.417 to 12.997) compared to other capital cities and rural areas (lowest to − 19.224 in ACT and highest to 59.575 in rural areas of New South Wales). Violin plots showing the statistical distribution of the vulnerability index in flood- and wildfire-affected areas (similar to Fig. 8) are displayed in Supplementary Figs. S10 and S11 with descriptions provided in Supplementary Note 5.

Spatial patterns of the three natural hazards (i.e., wildfire, earthquake, and flood) are displayed in Fig. 6A. Their patterns are substantially different. Wildfire appears predominantly in the north of Northern Territory, West Australia, and Queensland, and also spreads out along the eastern coast of Queensland. Earthquakes appear predominantly in the south of New South Wales, Victoria, South Australia and more widely in West Australia. Floods occur more frequently along with the hydrological network (e.g., rivers, streams, and creeks) spreading out in the whole Queensland and New South Wales, the northeast corner of South Australia, and the north of West Australia and Northern Territory. After overlapping the locations of natural hazards and the measures of the vulnerability index, we identify the most vulnerable areas with the highest level of risks (Fig. 6B and enlarged city maps in Fig. 7 and Supplementary Figs. S8 and S9). The identified names of the most vulnerable areas in hazard-affected areas are presented in Supplementary Table S10. We also enlarge the maps of capital cities to see more detailed locations of the vulnerable areas highly risky in earthquake (Fig. 7 with the place names presented in Supplementary Table S10). Statistically, the mean values of the vulnerability index in earthquake-affected areas of rural Queensland (8.177) and rural Northern Territory (8.638) are substantially higher than that in other states (e.g., 0.898 in urban areas of New South Wales and − 2.446 in ACT) (Fig. 8 and Supplementary Table S11).

**Figure 9:**
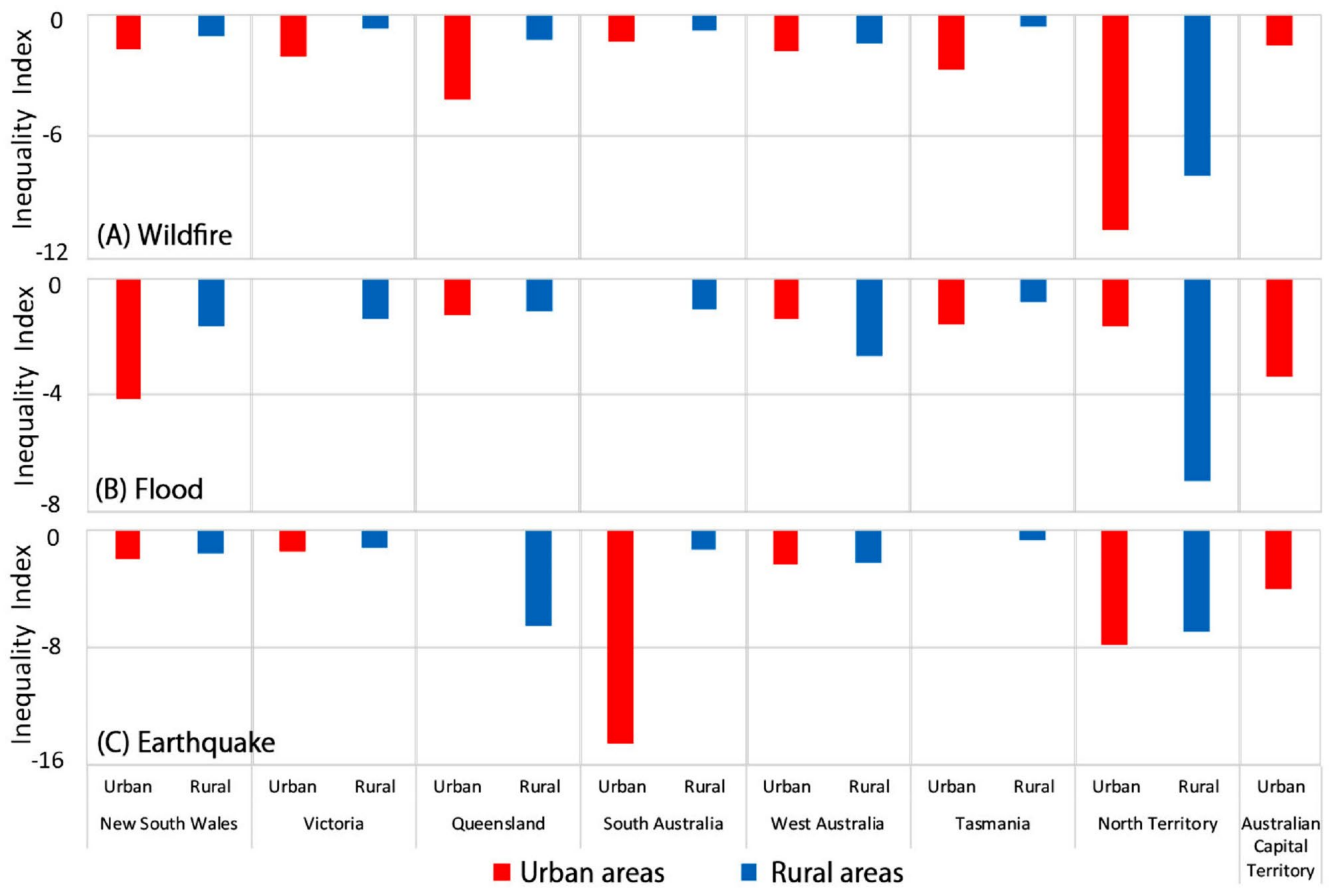
Inequality index of vulnerability in hazard-affected and non-hazard areas across urban and rural space. Here we only present the medium scenario ($k$=0.5) and the statistical summary of the inequality index on three levels of $k$ (0.25, 0.5, and 0.75) is provided in Supplementary Table S14. For wildfire (Fig. 9A), the inequality index in the urban areas of all states are larger than that their rural counterparts, reflecting vulnerability in wildfire-affected areas tends to be less equally distributed in urban than rural areas. Across states, the inequality of vulnerability in wildfire-affected areas is more obvious in Northern Territory (− 10.634 in urban areas vs. − 7.927 in rural areas) than other states. For flood (Fig. 9B), the magnitudes of the inequality index in the urban areas are larger than that its rural counterparts in New South Wales (− 4.168 vs. − 1.634), Queensland (− 1.269 vs. − 1.141), Tasmania (− 1.594 vs. − 0.798), but smaller in West Australia (− 1.376 vs. − 2.664) and Northern Territory (− 1.619 vs. − 6.985). It means that the inequality of vulnerability in flood-affected areas in urban areas of New South Wales, Queensland, Tasmania is more obvious than their rural counterparts. In particular, the inequality of vulnerability in flood-affected areas is more obvious in the rural areas of Northern Territory (− 6.985) and Sydney (− 4.168) than other regions (− 0.798 to − 3.387). For earthquake (Fig. 9C), the inequality of vulnerability in earthquake-affected areas in urban areas of all states is more obvious than their rural counterparts. In particular, the inequality of vulnerability in earthquake-affected areas in Adelaide (− 14.519), rural Queensland (− 6.533), and North Territory (− 7.903 vs. − 6.928) are more obvious than other regions.

Finally, we compare the inequality index of vulnerability in hazard-affected areas across urban and rural space by taking $k$ = 0.5 as the medium scenario (Fig. 9 with the statistical summary provided in Supplementary Table S14). For wildfire (Fig. 9A) and earthquake (Fig. 9C), the inequality index in the urban areas of all states are larger than that of their rural counterparts, reflecting vulnerability in hazard-affected areas tends to be less equally distributed in urban than rural areas. However, for flood (Fig. 9B), the inequality of vulnerability in hazard-affected areas in urban areas of New South Wales, Queensland, Tasmania is more obvious than their rural counterparts, but less obvious in other states. The statistical summary of the inequality index on three levels of $k$ (0.25, 0.5, and 0.75) is provided in Supplementary Table S14.

## Discussion

Using 41 indicators derived from multiple datasets, we constructed the first Australian nationwide fine-grained measures of vulnerability for urban and rural space, respectively. Methodologically, our study re-forged the Cutter’s vulnerability measuring framework with additional involvement of ‘4D’ built environment indicators, generating the measures of vulnerability in five themes—socioeconomic status, demographics and disability, minority status and language, housing type and transportation, and built environment. We further evaluated the inequality of vulnerability to three widely affected natural hazards (i.e., wildfire, flood, and earthquake) in Australia. We found that the inequality of vulnerability in hazard-affected areas in eight capital cities are more significant than that in their rural counterparts. The most vulnerable areas in capital cities were identified to be close to and/or on the urban fringe, where must be prioritized for adaptation; while the most vulnerable areas in rural regions are observed in the remote inland and culturally protected areas (e.g., indigenous protected areas in Northern Territory). The spatial patterns of vulnerability substantially vary across space and five themes. Our measures of vulnerability supplement the SEIFA indexes developed by ABS given it captures not only the demographic and socioeconomic profiles of the Australian population, but also features of housing, transport, and built environment. End users (e.g., government, public and private sectors, environmentalists, and academics) can be able to apply the theme-specific vulnerability index in various contexts and for distinct purposes (e.g., using Theme 4 of housing and transportation in the designation of housing insurance). Our results contribute to profiling Australia’s vulnerability and hazard risk assessment, and provide quantitative evidence for place-based risk reduction and sustainable urban–rural development.

There are observed consensuses between our measures of vulnerability and existing measures in the broader hazard literature in other geographic contexts, including Norway^14^, Nepal^15,16^, China^17^, Bangladesh^18^, Portugal^19^, India^20,21^, Brazil^22^, Colombia^23^, and Zimbabwe^24^. The indicators identified in existing studies (e.g., low-income, the elderly, minority, low education, unemployment) are also found to be relevant to vulnerability in our study (Supplementary Table S1). Furthermore, we identified a series of built environment indicators relevant to vulnerability (e.g., density of buildings) that were also found in the literature^6,7,14,17,25,27,30,46,46^, although existing studies do not measure built environment in the comprehensive way as our study did. The indicators in Theme 1–4 are similar to the indicators employed in the Social Vulnerability Index generated by the U.S. Centre for Disease Control^47^. In terms of the spatial pattern of vulnerability, we observed that the rural areas in the remote inland and urban areas close to and/or on the urban fringe tend to be more vulnerable. This finding is consistent with the observation in the U.S., and other countries where rural areas are more vulnerable than urban areas^4–6^. In face of the three natural hazards (i.e., earthquake, flood, and wildfire) in Australia, the inequality of vulnerability in hazard-affected areas in eight capital cities are more obvious than that in their rural counterparts. Such notable urban–rural disparity and inequality of vulnerability in turn imply the necessity of establishing two measuring systems for urban and rural space, respectively, given that certain indicators (e.g., indigenous population) may matter in rural but not urban areas.

Our findings suggest a number of policy implications in risk assessment and sustainable urban–rural development. First, governments and public authorities (e.g., Geoscience Australia) could directly adopt our nationwide measures of vulnerability in policy making and hazard mitigation planning; they can also employ our indicator-based measuring framework to quantify vulnerability over time and forge a longitudinal database to track the shift of vulnerability for their usage. Second, the delineation of the most vulnerable areas with a higher risk in natural hazards provides quantitative evidence for place-based risk assessment and locally-tailored hazard prevention and responses. For example, municipal governments should prioritise the risk reduction and adaptation in areas close to and/or on the urban fringe where residents are less socioeconomically advantaged and have less access to public facilities and social resources. Emergency agencies should consider the socially disadvantaged groups and have strategies to ensure that evacuation plans accommodate their needs. Third, to supplement the national disaster control strategies that have been developed^33,48,49^, our study yields new insights on the urban–rural disparity and inequality that should be integrated into spatially explicit designation of risk coping strategies to improve the sustainability of urban–rural development. The planning process should be multisectoral and integrate all issues relevant to the current stage of development, especially in the rapid growth of peri-urban areas in Australia.

There are some limitations in our study to guide through future research direction. First, our methods employed the unweighted additive model to generate the final vulnerability index. More refinements are necessary by utilising the weighted model and developing a defensible weighting scheme to determine relative weights. It can be based on experts’ suggestions, voting from the public, or workshop with professionals^6^. Also, future studies can test out the multiplicative approach and/or a combined additive-multiplicative approach to construct vulnerability index so that the importance of each component of vulnerability index would be distinguished to some degree. Second, more attempts are needed to develop longitudinal measures of vulnerability across censual years through robust and replicable experiments. In that case, we would be able to track the change of vulnerability over time and unveil the potential drivers of such changes. Third, our vulnerability index can be applied for risk assessment of other natural hazards (e.g., storm surge, cyclones and, landslides) by simply using our vulnerability measures and replacing the measures of our selected three hazards with other types of hazards. It can be further coupled with economic loss data to further examine those individual indicators that are the most important contributors to economic losses. Our measures of vulnerability are publicly accessible via our project public repository. They can be readily used by government, planning authorities, and other end-users (e.g., researchers and students) for specific purposes. Fourth, the indicators identified in our study demonstrate their strong relevance to vulnerability, however, they not reveal the underlying causes of that vulnerability. Thus, the interpretation of vulnerability in the hazard coping strategies should be dealt with great cautions.

## Conclusion

In summary, we contribute the first nationwide study that measures vulnerability across Australia and examines the inequality of vulnerability in the hazard-affected areas across eight capital cities and their rural counterparts. Our key findings include that the inequality of vulnerability in hazard-affected areas in eight capital cities are more significant than that in their rural counterparts while the most vulnerable areas in capital cities largely appear in peri-urban regions. The most vulnerable areas in rural regions are observed in the remote inland and culturally protected areas. Methodologically, we re-forge the Cutter framework by additionally involving the ‘4D’ quantification of built environment, and enrich the existing literature by establishing two measuring systems of vulnerability for urban and rural areas, respectively. Our analytical framework is readily to be reapplied to other geographic contexts and evaluate the vulnerability to other natural hazards. We enhance the Cutter’s framework and the categorisation of four themes employed by the US CDC with five themes—(1) socioeconomic status; (2) demographics and disability; (3) minority and languages; (4) housing characteristics; and (5) built environment—the associated measures are the first step in paving the way for both urban and rural areas to develop smart evidenced based policy around climate-resiliency and steer their respective urban and rural development toward environmental sustainability. Our findings on urban–rural disparities and the scope and scale of the inequality of vulnerability in response to natural hazards provide spatially explicit new evidence for government, the public and private sectors, urban planners and policy makers to profile hazard risks and forge risk coping strategies in Australia.

## Method

### Measures of the vulnerability index

The indicators used for measuring the vulnerability index in Theme 1–4 (Supplementary Table S2) were retrieved from the Census of Population and Housing via Table Builder Portal from the Australian Bureau of Statistics^50^. The census data was retrieved at the Statistical Area level 1 (SA1) as the smallest census unit. SA1 areas generally have a population of 200 to 800 people, and an average population of about 400 people^51^. SA1 areas in remote and rural regions generally have smaller populations than those in urban areas. There are 57,523 SA1 areas in total in Australia; amongst those, 1313 SA1 areas with population less than 50 were excluded in our analysis because these small population numbers were randomly assigned by ABS data privacy protection. The remaining 55,218 SA1 areas were used in the analysis.

The indicators in Theme 5 were derived from multiple data sources. First, Points of Interest (PoI) data originally retrieved via the Open Street Map were provided by Australian Urban Research Infrastructure Network^52^. The PoI data contains the locations (X, Y coordinates) of + 100 types of places and we reclassified them to a total of 24 major types (Supplementary Table S14). They were used to calculate the nearest distance of a SA1 area (the centroid of a SA1) to a particular place using the ‘*nearest’* function in ArcGIS Pro 2.8. Second, the digital cadastral data from Department for Infrastructure and Transport contains the nationwide road and railway networks^53^. We selected five types of drivable roads from the road network data—‘freeways/motor ways’, ‘high ways’, ‘secondary ways’, ‘local connector roads’ and ‘street/local roads’—used for measuring the distance from a place to the nearest road segment. Such distances were calculated in ArcGIS Pro 2.8 using the ‘*nearest’* function. Third, the Sentinel-2 satellite imagery retrieved via Google Earth Engine (2020) were used to calculate the normalized difference built-up index (NDBI) to indicate the coverage of built-up areas (code provided in Supplementary Note 6)^54^. Sentinel-2 carries a multispectral imagery with a swath of 290 km. The imagery provides a versatile set of 13 spectral bands spanning from the visible and near infrared to the shortwave infrared, featuring four spectral bands at 10 m, six bands at 20 m and three bands at 60 m spatial resolution. NDBI highlights urban areas where there is typically a higher reflectance in the shortwave-infrared region, compared to the near-infrared region (Google Earth Engine, 2020). The NDBI value ranges from − 1 to + 1. Negative values represent water bodies whereas the higher positive values represent the bigger build-up areas. Fourth, the land use data from the Department of Agricultural, Water and the Environment contained ten types of land use, including commercial, education, hospital, industrial, parkland, primary production, residential, transport, water, and other land use^55^. Then, we used Simpson’s index to measure land-use diversity as Eq. 1^56^:

1 $$Simpson^{\prime}s Index = 1 - \sum \frac{{n_{i}^{2} }}{{N^{2} }}$$

where ${n}_{i}$ indicates the total number of areas in one SA1 for land use type i; N is the total area of all land use types, i is the types of land use classified into ten. Simpson’s Index ranges from 0 (minimum diversity) to 1 (maximum diversity). Equation 1 was also used to calculate the ethnicity diversity and housing diversity based on the census data (Supplementary Table S2). In the end, we derived a total of 41 indicators, including four indicators in four themes After normalizing some indicators via log-transformation, all 41 indicators were transferred to be normally distributed (Supplementary Table S2).

We then employed the principal component analysis (PCA) to generalize the underlying structure of the 41 indicators and extracted the principal components that were used to construct the overall vulnerability index. PCA allows for a robust and consistent set of variables that can be monitored over time to assess any changes in overall vulnerability^57^. The PCA used to generate the standardising principal component scores is expressed as^57^:

2 $${PC}_{SA1}=\sum_{i=1}^{j}\frac{{L}_{i}}{\sqrt{\mu }}\times {X}_{i, SA1}$$

where ${PC}_{SA1}$ denotes the raw principal component score for one SA1; ${X}_{i, SA1}$ denotes the standardized indicator of the $i$-th indicator for the SA1; ${L}_{i}$ is the loading for the $i$-th indicator; $\mu$ denotes the engenvalue of the principal component; $j$ is the total number of indicators in that principal component.

We conducted PCA for urban and rural areas, generating a total of 10 PCs and 11 PCs, respectively. The overall vulnerability index ($VI$) was calculated by using an evenly-weighted arithmetic (additive) aggregation procedure^6^:

3 $$VI=\sum_{n=1}^{p}{PC}_{n,SA1}$$

where ${PC}_{n,SA1}$ is the eigenvalue of that PC in one SA1 and $p$ is the total number of PCs extracted in that SA1.

### Validation and sensitivity analysis

We conducted a sensitivity analysis by running the PCA for urban space (eight capital cities) to extract different number of PCs and testing whether the outcome measure of vulnerability is sensitive to the selection of PCs. In addition to the first scenario (extracting 10 PCs) that we did previously, we generated two more scenarios with 12 and 17 PCs extracted to explain the 74.58 and 84.69% of data variance, respectively (Supplementary Table S15). We then compared these two scenarios with the first scenario (10 PCs) by conducting a pairwise Pearson’s correlation^58^ and the one-sample t-test^59^ to assess the sensitivity of PC selection on the construction of VI. The result of the Pearson’s correlation (Supplementary Table S16) shows that VI generated by three scenarios are highly correlated with the coefficient above 0.773 (*p* < 0.01). The result of the T statistics (Supplementary Table S17) indicates that the means of VI generated by three scenarios have insignificant differences (*p* > 0.1). It means that the VI generated by the first scenario (based on 10 PCs) is representative thus we select the first scenario to measure VI for the purpose of simplification. To validate our measures of VI, we also conducted a pairwise Pearson’s correlation analysis between VI and four indices of SEIFA developed by ABS, including Index of Relative Socio-Economic Disadvantage (IRSD), Index of Relative Socio-Economic Advantage and Disadvantage (IRSAD), Index of Education and Occupation (IEO), and Index of Economic Resources (IER)^43^. The result shows that VI in Theme 1 (socioeconomic status) is highly correlated with the four indices of SEIFA (Supplementary Fig. 12) but VI in other themes are not. It means that our measures of VI capture multiple dimensions of vulnerability that SEIFA is not able to fully cover.

### Estimating natural hazards

#### Hazard 1: Earthquake

Earthquake data was retrieved from the National Seismic Hazard Assessment for Australia (NSHA18) developed by Geoscience Australia^60^. This NSHA18 dataset contained time-independent, mean seismic design values which were calculated on rock sites (Standards Australia’s AS1170.4 Soil Class) for the geometric mean of the 5% damped response spectral accelerations, Sa(T), for different timespans (e.g., from 0.1 to 4.0 s)^60^. The hazard values were estimated across the Australian continent using a uniformly-spaced 15 km grid. Hazard curves and uniform hazard spectra were also calculated for key localities at the 10 and 2% probability of exceedance in 50-year hazard levels. We selected the seismic map with Sa(T) of 0.2 s at the 2% probability of exceedance in 50-year hazard levels given that it had a wider range of estimated earthquake probabilities (originally ranging from 0 to 71%) compared to other seismic maps with different parametric settings. We then utilised the ‘reclassify’ function in ArcGIS Pro 2.8 to re-categorise the earthquake probability to three risk levels based on the equal interval (i.e., 0–23.7% as low risks coded as 1, 23.7–47.3% as medium risks coded as 2, and 47.3% to 71% as high risks coded as 3) (high (Fig. 6A-1).

#### Hazard 2: Wildfire

Fires in Australia’s forests 2011–16 (2018) dataset was collected from the Australian Bureau of Agricultural and Resource Economics and Sciences^61^. It contains the extent and frequency of planned and unplanned fires that occurred in the five financial years between July 2011 and June 2016, and reported from multiple fire area datasets contributed by state and territory government agencies. This fire dataset is in raster format with a resolution of 100 m. It has a key attribute ‘TOTAL_X_BURNT’ indicating the number of times burnt in each cell (originally ranging from 0 to 5), which was reclassified to three levels of risks using ‘reclassify’ function in ArcGIS Pro 2.8 based on the equal interval method (i.e., 0–1.67 as low risks coded as 1, 1.67 to 3.34 as medium risks coded as 2, and 3.34 to 5 as high risks coded as 3) (high (Fig. 6A-2).

#### Hazard 3: Flood

Flood data at a global scale was retrieved as a collection of flood maps from Joint Research Centre (JRC) Data Catalogue, European Commission^62^, depicting flood prone areas for river flood events of different magnitudes (e.g., from 1-in-10-year to 1-in-500-year). We did not use the historical flood map here as the extend of historical flood events was too small and sporadic to observe in the national scale. Instead, the JRC flood maps were estimated and simulated using hydrological and hydrodynamic models, driven by the climatological data of the Global Flood Awareness Systems (GloFAS). After tailoring to the Australian scale, they comprise most of the geographical Australia (excluding external territories such as Christmas Island) and all the river basins in the Australian mainland and the state of Tasmania. Flood maps are in raster format (GEOTIF) with a grid resolution of 30 arcseconds (approximately 245 m after projection). We selected three flood maps (i.e., 1-in-10-year, 1-in-100-year, and 1-in-500-year) and each map has binary attributes with 0 indicating no flood cells and 1 indicating flooded cells. We then overlapped these three maps by the *‘raster calculator’* function in ArcGIS Pro 2.8, generating the final flood risk map with attribute values ranging from 0 indicating no flood risk and 1–3 indicating flood risk levels from low to high (Fig. 6A-3).

### Inequity metrics

We utilised the inequity index developed by Gluschenko^63^ to evaluate the inequality of vulnerability in hazard-affected areas across urban and rural space. The inequity index has the advantage to characterise distributions of environmental hazards^63^, as it allows the input variable (i.e., vulnerability) to be negative values and also it allows the adjustment of the parameter $k$ in Eq. (4) to reflect the non-linearity of marginal damages caused by three types of natural hazards^64^. The lower value of $k$ corresponds to a higher marginal damage of the hazard $x$, resulting in a higher inequality index value for a given unequal distribution. When $k$ approaches zero, the inequality index would be close to zero. For the vector of VI, the inequality index can be expressed as^63^:

4 $$I\left(x\right)= -\frac{1}{k}Ln\frac{1}{N}\sum_{n=1}^{N}{e}^{k\left[\overline{x }-{x}_{n}\right]}, for k<0$$

where $\overline{x }$ is the mean of the vulnerability in each urban or rural SA1 area and $k$ is a parameter indicating the degree to which inequality in the distribution is undesirable due to increasing marginal damage^63^.

Since there is no consensus on the selection of $k$^65–67^, the literature typically presents results for a range of values^64^. Thus, we selected three possible values for $k$(0.25, 0.5 and 0.75) in the calculation of the inequality index. Existing studies use inequality measures for which the elasticity is a constant. For the measure of the inequality index, however, this elasticity, ${x}_{n}$, is a function of $x$. To present results for a range of $k$ that generates elasticities comparable to those in the existing literature, we first identify a value of $k$ that is consistent with a given constant elasticity. To establish a correspondence between an elasticity and a vector of elasticities $x$, we use the below approach of choosing the value of $k$ that minimizes the sum of squared differences between the individual elasticities:

5 $$k\left(\beta \right)=-{\mathrm{arg}}_{\widehat{k}}\mathrm{min}\left\{{\left[\widehat{k}x-\beta \right]}^{^{\prime}}\left[\widehat{k}x-\beta \right]\right\}=-\frac{\beta \sum_{n=1}^{N}{x}_{n}}{\sum_{n=1}^{N}{x}_{n}^{2}}$$

where $\beta$ denotes the given constant elasticity; $N$ denotes the total number of SA1 areas; ${x}_{n}$ is the vulnerability score of a given SA1 area $n$. Here we employed three levels of inequality aversion—low $k$(0.25), moderate $k$(0.50), and high $k$(0.75)—representing different inequality aversions to calculate the inequality index presented in Supplementary Table S13.

### Ethics statement

This study did not receive nor require ethics approval, as it does not involve human & animal participants.

## Supplementary Information

Supplementary Information.

## Data Availability

Vulnerability data generated in this study is available to visualise and download in the project website: https://projects.iq.harvard.edu/chinadatalab/AU_vulnerability. All other data used in this study are publicly available. More specifically, the data used to measure vulnerability are from 1) Census of population and housing, Table Builder Portal, Australian Bureau of Statistics, 2016 (https://www.abs.gov.au/statistics/microdata-tablebuilder/tablebuilder); 2) Open Street Map – Points of Interest (Australia), Australian Urban Research Infrastructure Network, 2020 (https://data.aurin.org.au/dataset/osm-osm-points-of-interest-2020-na); 3) Digital cadastral database, Department for Infrastructure and Transport, Australian Government, 2020 (https://data.gov.au/dataset/ds-sa-4cc17ac3-ce49-4525-971b-6122023b8937/details); 4) Land use data, Department of Agricultural, Water and the Environment, Australian Government, 2016 (https://www.awe.gov.au/abares/aclump/land-use/data-download); 5) Sentinel-2 satellite imagery, Google Earth Engine, Google 2020 (https://developers.google.com/s/results/earth-engine/datasets?q=LANDSAT). The hazard data are from 6) Fires in Australia’s forests 2011–16 (2018), Australian Bureau of Agricultural and Resource Economics and Sciences, 2018 (https://data.gov.au/dataset/ds-dga-c0cd6f5a-6b0f-4cd0-8980-1ca5b2c31006/details); 7) Data Catalogue-Global Flood Awareness System, Joint Research Centre, European Commission, 2020 (https://data.jrc.ec.europa.eu/collection/id-0069); 8) The 2018 National Seismic Hazard Assessment for Australia, Geoscience Australia, 2018 (https://data.gov.au/dataset/ds-neii-683d597a-d639-4020-b056-0cb187b717ca/details?q=).

## Code availability

Code used to retrieve the imagery from Google Earth Engine is provided in Supplementary Note 6. Data analyses are conducted in Excel, SPSS, ArcGIS Pro 2.8, and R with code provided in the project public repository: https://projects.iq.harvard.edu/chinadatalab/AU_vulnerability.
