# Supplementary material for: Urban–rural disparity of social vulnerability to natural hazards in Australia
Source: Sci Rep. 2022 Aug 11;12:13665. doi: 10.1038/s41598-022-17878-6 (PMC9372054; doi:10.1038/s41598-022-17878-6)
Supplement: Supplementary file 1 — Supplementary Information. [file 41598_2022_17878_MOESM1_ESM.pdf]

Supplementary Materials

Table of Contents

Table 1. 3
Note 1 (Study area) 6
Table S2. 7
Note 2 (Identified indicators for Theme 2 to 5) 10
Table S3. 12
Table S4. 13
Table S5. 14
Table S6. 15
Note 3 (Details of the most vulnerable areas in capital cities) 16
Figure S2. 17
Figure S3. 18
Figure S4. 19
Figure S5. 20
Figure S6. 21
Table S7. 22
Table S8. 23
Figure S7. 24
Table S9. 24
Figure S8. 25
Figure S9. 26
Note 4 (Details of the most vulnerable and highly risky areas in eight capital cities) 27
Table S10 27
Table S11 28
Note 5 ((Details of the most vulnerable areas highly risky in flood and wildfire) 28
Figure S10. 29
Figure S11. 30
Table S12 30
Table S13 31
Table S14 32
Table S15 33
Note 6 (Generating NDBI) 34

Table S16..35
Table S17..37
Table 18. 37
Figure S12. 37
Supplementary Reference 38

Table 1. Vulnerability concepts, linkage to hazards and expected effect

Concept	How it links to human responses to hazard	Source	Expected effect (+/-) on vulnerability
Employment	Unemployed persons do not have employee benefits that provide income and health insurance during hazards.	Holand, Lujala et al. 2011, Aksha, Juran et al. 2019, Cumberbatch, Drakes et al. 2020	(+)
Income	Low-income families are less likely to have the income or assets needed for preparedness and recovery.	Cutter, Mitchell et al. 2000, Wigtil, Hammer et al. 2016, Aksha, Juran et al. 2019	(+)
Education	People with lower levels of education are less likely to have access to and act upon varied hazard information from preparation to recovery.	Tierney 2011, Wigtil, Hammer et al. 2016, Krishnan, Ananthan et al. 2019	(+)
Occupation	Those who work in the low-skilled sectors are more likely to lose their jobs in cases of hazards, which in turn contributes to slower recovery.	Holand, Lujala et al. 2011, Cutter, Ash et al. 2014, Kirby, Reams et al. 2019	(+)
Elderly	Elderly, especially those who live alone, who have physical, sensory, or cognitive challenges are likely to be more difficult to evacuate and recover during and after hazards.	Cutter, Mitchell et al. 2000, Peek-Asa, Ramirez et al. 2003, Rosenkoetter, Covan et al. 2007, Holand, Lujala et al. 2011	(+)
Children	Children, especially in the youngest age groups, cannot protect themselves during hazards because lack the necessary resources, knowledge, or life experiences to cope with the situation.	Cutter, Mitchell et al. 2000, Cutter, Boruff et al. 2003, Martin, Bush et al. 2006, Cumberbatch, Drakes et al. 2020, Drakes, Tate et al. 2021	(+)
Median age	Both high and low values of median age conceivably can indicate high vulnerability.	Cutter, Boruff et al. 2003, Burton and Cutter 2008, de Oliveira Mendes 2009, Wigtil, Hammer et al. 2016	(+/-)
Economic dependency	Families with large numbers of dependents may face difficulties to outsource care for dependents, and thus may need to balance work responsibilities and care for family members which could affect the resilience to and recovery from hazards.	Chen, Cutter et al. 2013, Krishnan, Ananthan et al. 2019, Drakes, Tate et al. 2021	(+)
Gender	Women may couple with household responsibilities which could be more vulnerable to recover from hazards compared to men.	Aksha, Juran et al. 2019, Kirby, Reams et al. 2019, Krishnan, Ananthan et al. 2019, Cumberbatch, Drakes et al. 2020	(+)
Family composition	More persons within a household could increase the economic burden of evacuation and recovery from hazards.	De Oliveira Mendes 2009, Akinola, Adegoke et al. 2019, Drakes, Tate et al. 2021	(+)
Social dependency	Caring for children may impact parents' preparation of evacuation and recovery. Parents may lose time and money caring for children when daycare facilities are affected during hazards.	Chen, Cutter et al. 2013, Nicholson, Attal-Juncqua et al. 2021; De Oliveira Mendes 2009, Chen, Cutter et al. 2013	(+)

Need assistance	Special needs populations may encounter difficulties during evacuation. They could also be invisible in communities and societies during the recovery phase.	Flanagan, Gregory et al. 2011, Chen, Cutter et al. 2013, Cutter, Ash et al. 2014, Akinola, Adegoke et al. 2019, Aksha, Juran et al. 2019	(+)
Marital status	Single/separated adults earn less than those who are currently married. This economic gap could increase their vulnerability to hazards recovery. The emotional support from partners may also reduce individuals' vulnerability during hazards.	Akinola, Adegoke et al. 2019, Drakes, Tate et al. 2021	(+)
Minority (migrants/immigrants)	Immigrants may be unfamiliar with local resources which could increase the difficulties to navigating the resource and receiving updated information on disaster preparedness and recovery. Immigrants are likelier to rely on local social networks which may impact their ability to evaluate to other cities and states.	Cutter, Boruff et al. 2003, Burton and Cutter 2008, Schmidtlein, Deutsch et al. 2008, Holand, Lujala et al. 2011, Aksha, Juran et al. 2019	(+)
English	Limited English proficiency could impact individuals' ability to access updated information on hazards.	Cutter, Ash et al. 2014, Wigtil, Hammer et al. 2016, Berke, Yu et al. 2019, Drakes, Tate et al. 2021; Solangaarachchi, Griffin et al. 2012, Wigtil, Hammer et al. 2016, Akinola, Adegoke et al. 2019	(+)
Ethnicity	Being ethnic minority is associated with lower access to updated information on hazards and lower allocations of hazards-related assistance.	Cutter, Boruff et al. 2003, Burton and Cutter 2008, Chen, Cutter et al. 2013, Wigtil, Hammer et al. 2016, Drakes, Tate et al. 2021	(+)
Housing structure	People living in multi-unit apartments/houses are vulnerable to overcrowding when funneled into a limited number of exit stairwells during hazard evacuation.	Cutter, Boruff et al. 2003, Flanagan, Gregory et al. 2011, Berke, Yu et al. 2019	(+)
Paying mortgage	House owners need to continue to pay mortgage even if their home is destroyed or unliveable due to a disaster. House owners have to repair or rebuild their house at their own expense which could increase their burden during recovery.	De Oliveira Mendes, 2009	(+)
Mobile living	Mobile homes are not designed to withstand severe weather or flooding which are easily destroyed and less resilient to hazards. They are also frequently found outside of metropolitan areas which may not be readily accessible by interstate highways or public transportation.	Cutter, Mitchell et al. 2000, Cutter, Boruff et al. 2003, Burton and Cutter 2008, Wigtil, Hammer et al. 2016, Drakes, Tate et al. 2021	(+/-)
Car ownership	Transportation out of an evacuation zone is problematic for people who do not have access to a vehicle to evaluate during hazards.	Flanagan, Gregory et al. 2011, Cutter, Ash et al. 2014, Wigtil, Hammer et al. 2016, Drakes, Tate et al. 2021	(+)
Internet access	Households with no internet connection may have difficulties receiving updated information on hazards.	Solangaarachchi, Griffin et al. 2012, Cutter, Ash et al. 2014	(+)

Rental	People that rent do so because they are either transient or do not have the financial resources for homeownership. They often lack access to information about financial aid during recovery.	De Oliveira Mendes 2009, Chen, et al. 2013, Aksha, et al. 2019; Burton and Cutter 2008, Solangaarachchi et al. 2012, Kirby, et al. 2019	(+)
Housing type	The poor physical and social living conditions in overcrowded households could contributors to increased social vulnerability during hazard recovery.	De Oliveira Mendes 2009, Flanagan, Gregory et al. 2011, Berke, Yu et al. 2019	(+)
Distance to cities	Cities could be more vulnerable to hazards due to building density; they could also be less vulnerable due to better access to resources and transportation for evacuation and recovery.	Mainali and Pricope 2017, Krishnan, Ananthan et al. 2019	(+/-)
Access to public transits	Access to public transportation plays an important role in rapid evacuation, especially for those who do not have access to a vehicle.	Flanagan, Gregory et al. 2011, Kirby, Reams et al. 2019	(+)
Density of commercial places	Easy access to commercial places could help with hazard preparedness. Many commercial places are also used as shelters which may also help with hazard recovery.	Burton and Cutter 2008, de Oliveira Mendes 2009, Cutter, Ash et al. 2014, Krishnan, Ananthan et al. 2019	(+)
Density of public service	Long distance to public services during hazards may increase hazards related damage.	De Oliveira Mendes 2009, Kirby, Reams et al. 2019	(+)
Road access	Long distance to roads could increase the difficulties of evacuation.	Cutter, Ash et al. 2014, Kumar and Bhattacharjya 2020	(+)
Density of hospitals	Hazards may cause injuries and other health issues. Long distance to healthcare service decrease the possibility to obtaining efficient medical treatment.	Burton and Cutter 2008, de Oliveira Mendes 2009, Holand, Lujala et al. 2011, Chen, Cutter et al. 2013, Krishnan, Ananthan et al. 2019	(+)
Density of buildings	The higher the density of building shows the higher concentration of people, economic activity, traffic, and built structures, which lead to greater potential losses resulting from hazards.	Cutter, Boruff et al. 2003, Burton and Cutter 2008, Holand, Lujala et al. 2011, Chen, Cutter et al. 2013	(+)
Density of roads and railways	Length of roads and railways represents lifelines and an ability to move people away from an area, which indicates a level of evacuation potential.	Cutter, Ash et al. 2014, Kirby, Reams et al. 2019, Cumberbatch, Drakes et al. 2020	(-)
Street design	Streets with stormwater drainage, water, and sewer are considered to be more resilient from hazards.	Berke, Yu et al. 2019, Prosdocimi and Klima 2020	(+)

Note 1 (Study area)

Australia is the largest developed country in the Southern Hemisphere, with a total population of nearly 26 million and a total area of around 7.61 million square kilometres (Australia Bureau of Statistics (ABS), 2022). Australia is highly urbanised, with over 80% of its population living in cities. The nation's capital city is Canberra, also known as the Australian Capital Territory (ACT), and the other states/territories are (the state capital cities are listed in brackets): New South Wales (Sydney), Victoria (Melbourne), Queensland (Brisbane), West Australia (Perth), South Australia (Adelaide), Tasmania (Hobart), and Northern Territory (Darwin). According to the Greater Capital City Statistical Area Structure (ABS, 2016), each state is divided into a greater capital city area and the remaining regional/rural area. For instance, the State of New South Wales (NSW) is divided into the Greater Sydney Area and Rest of NSW. In this paper, we simplified the terminology using Sydney and Beyond Sydney to represent Greater Sydney and the Rest of NSW, respectively, in later analysis (Figure S1); such simplification also applied to other states/territories with the exception of ACT having only the capital city for the whole territory. Australia has a long history of natural hazards including wildfires (bushfires), floods, earthquakes, storms, cyclones and landslides. The impact associated with hazards also varies and can range from frequent moderate impacts (e.g. bushfires) through to rare but potentially catastrophic impacts (e.g. earthquakes). We selected three types of natural hazards (i.e., wildfires, floods and earthquakes) as wildfires and floods are the top two most notable and common natural hazards in Australia, and earthquakes have the wider impacts on both inland and coastal regions compared to storm surges and cyclones that happened more frequently but with impacts predominantly on coastal regions.

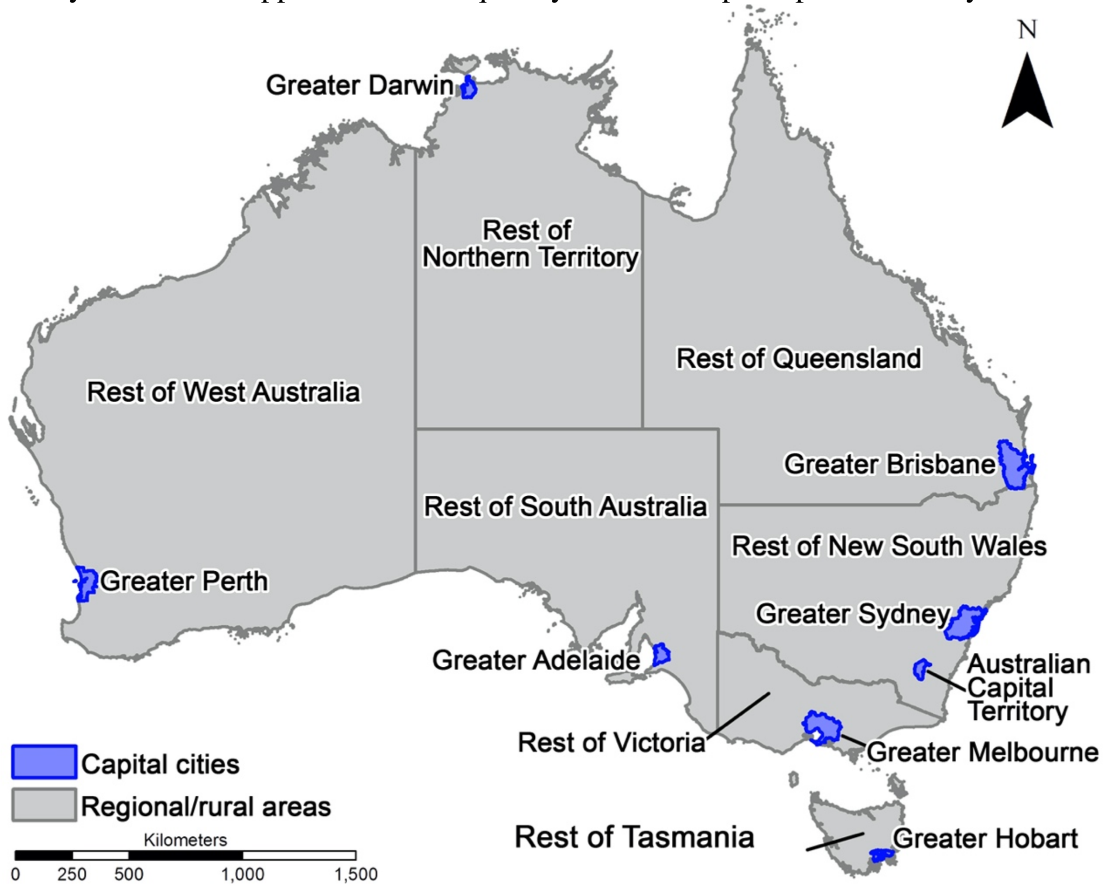

Figure S1. Geographic contexts of Australia

Table S2. Indicators used in the principal component analysis

Indicator	Definition
**Theme 1: Socioeconomic status** [A]	
Unemployed (%)	Unemployed people over the total labour force
Low income (%)	Households with weekly income less than $800 over the total households
Low education (%)	People with the highest education level at high school or lower
Non-MPA occupation (%)	People with occupation not in manager, profession, and clerical and administration over the total pop
**Theme 2: Demographics & disability** [A]	
Age above 65 (%)	People with age at or above 65over the total population
Age 0-18 (%)	People with age at 0 to 18 over the total population
Median age (%)	Weighted average age across all age groups
Not labour force (%)	People not in the labour force over the total population
Female (%)	Female over the total population
Multi-family HH (%)	Households with two or more families over the total households
Need child care (%)	People with need for child care over the total population
Need assistance (%)	People who disabled and need assistance over the total population
Children (%)	People who had children over the total population
Not married (%)	People not married over the total population
**Theme 3: Minority & languages** [A]	
Minority (%)	People born overseas (outside of Australia) over total population
Bad English (%)	Non-English-speaking people who speaks English 'not well and not at all' over the total population
Non-English speaker (%)	People with non-English native language over the total population
Indigenous (%)	Aboriginal people and Torres Strait Islanders over the total population
Ethnic diversity (index)	The Simpson's index for ethnic diversity at SA1 level, calculated as: $ED = 1 - \sum \frac{{n_i}^2}{N^2}$, where n_i= the number of people belonging to group i (i=5 cultural groups); N = total number of population, i = cultural groups by birthplace (continental): Oceania; Europe; Middle east and Africa; Asia; America. ED score ranges from 0 (minimum diversity) to 1 (maximum diversity)
**Theme 4: Housing characteristics** [A]	
Multi-unit houses (%)	Multi-unit dwellings (including semi-detached, unit, flat, apartment) over the dwellings
Low mortgage (%)	People who own houses and paying low mortgage (less than 2,000 AUD per month) over the total population
Retired village (%)	Retired villages over total dwellings

Mobile living (%)	Households living in mobile places (e.g., caravan park, camping sites, manufactured houses) over the total households
No car (%)	Dwellings without car over total dwellings
No internet (%)	Dwellings without internet access over the total dwellings
Renters (%)	Renters over the total population
Rent (%)	Rented dwellings over the total dwellings
Small houses (%)	Dwellings with one bedroom over total dwellings
**Theme 5: Built environment**	
Distance to cities (m) [B]	Distance to the nearest city/town centre (measured by the ‘near’ function in ArcGIS Pro 2.8)
Distance to public transits (m) [B]	Distance to the nearest public transit stop, including the stop of all types of public transits such as bus, tram, railway, and ferry stations (measured by the ‘near’ function in ArcGIS Pro 2.8)
Distance to commercial (m) [B]	Distance to the nearest commercial place, including a supermarket, convenience, hairdresser, clothes, pharmacy, beverages, bakery, butcher, car dealership, department store, beauty shops, laundry, bicycle shops, gift shops, newsagent, furniture shop, greengrocer, jeweller, florist, outdoor shop, bookshop, shoe shop, sports shop, chemist, mobile phone shop, cinema, optician, computer shop toy shop, general, marketplace, mall, and video shop (measured by the ‘near’ function in ArcGIS Pro 2.8)
Distance to public service (m) [B]	Distance to the nearest public service, including a town hall, embassy, public building, post box, telephone, post office, police station, fire station, community centre, and library (measured by the ‘near’ function in ArcGIS Pro 2.8)
Distance to roads (m) [C]	Distance to the nearest road network (measured by the ‘near’ function in ArcGIS Pro 2.8)
Distance to healthcare (m) [B]	Distance to the nearest healthcare facility, including hospital, Doctors, dentist, nursing home (measured by the ‘near’ function in ArcGIS Pro 2.8)
Diversity of land use (index) [D]	The Simpson’s index for land-use diversity at the SA1 level, calculated as: $LUD = 1 - \sum \frac{{n_i}^2}{N^2}$, where n_i=total number of areas in one SA1 for land use type i; N = total area of all land use types, i=types of land use classified into ten types, including commercial, education, hospital, industrial, parkland, primary production, residential, transport, water and other land use. LUD score ranges from 0 (minimum diversity) to 1 (maximum diversity).
Diversity of housing (index) [A]	The Simpson’s index for housing heterogeneity at SA1 level, calculated as: $HD = 1 - \sum \frac{{n_i}^2}{N^2}$, where n_i=total number of a certain type of dwellings in that SA1 i; N = total number of dwellings in that SA1, i=types of dwellings classified into five types, including separated house, semi-separated (detached), flat, cabin, caravan. HD score ranges from 0 (minimum diversity) to 1 (maximum diversity).
Density of buildings (m^2/km^2) [B]	Building areas over the total area of a SA1

Density of roads and railways (m/km^2) [C]	Total length of roads and railways over the total area of a SA1
Index of built-up areas [E]	The normalized difference built-up index ranging from -1 to 1; the higher positive values represent the bigger build-up areas in a SA1
Street design (count per km^2) [B]	Number of street facilities (e.g., benches, streetlamps, drinking water pumps) over the total area of a SA1
Connectivity design (count per km^2) [B]	Number of traffic connectivity (e.g., crossing, roundabout, motorway junctions, slipway, stop signs, traffic signals, and turning circles) over the total area of a SA1

Note:
A Census of population and housing, Table Builder Portal (Australian Bureau of Statistics, 2016
https://www.abs.gov.au/statistics/microdata-tablebuilder/tablebuilder
B Open Street Map – Points of Interest (Australia), Australian Urban Research Infrastructure Network, 2020
https://data.aurin.org.au/dataset/osm-osm-points-of-interest-2020-na
C Digital cadastral database, Department for Infrastructure and Transport, Australian Government, 2020
https://data.gov.au/dataset/ds-sa-4cc17ac3-ce49-4525-971b-6122023b8937/details
D Land use data, Department of Agricultural, Water and the Environment, Australian Government, 2016
https://www.awe.gov.au/abares/aclump/land-use/data-download
E Sentinel-2 satellite imagery, Google Earth Engine, Google 2020
https://developers.google.com/s/results/earth-engine/datasets?q=LANDSAT

Note 2 (Identified indicators for Theme 2 to 5)

In Theme 2 of demographics and disability (Fig 2), identified indicators for urban space include age above 65 (α= 0.942), median age (α= 0.924), not in labour force (α= 0.806) and needing assistance (α= 0.706). While there are additional indictors identified for rural space, including age 0-18, need child care, indigenous, multi-family households, and female composition. These indicators explain 4.125% and 8.281% of the variation for urban and rural areas, respectively (Supplementary Table S3 and S4). It is consistent with the literature concluding that two demographic groups are most affected by disasters, children and the elderly (Cutter, Mitchell et al. 2000, Cutter, Boruff et al. 2003, Martin, Bush et al. 2006, Cumberbatch, Drakes et al. 2020, Drakes, Tate et al. 2021; Peek-Asa, Ramirez et al. 2003, Rosenkoetter, Covan et al. 2007, Holand, Lujala et al. 2011). The preponderance of children (correspondingly households needing child care) and the elderly, together with populations not in labour force in the community load positively on this dimension. Indigenous population is only identified as the indicator for vulnerability in rural areas, possibly explained by the fact that regional and remote areas in Northern Territory and central Australia (e.g., culturally protected areas) inhabit a relatively larger number of indigenous and aboriginal populations compared to urban space.

In Theme 3 of minority and languages (Fig 2), indicators for measuring vulnerability in urban areas include non-English speaker (α= 0.909), minority (α= 0.859), and bad English (α= 0.835); while rural areas include indicators minority, bad English and ethnic diversity which are similar to indicators in urban areas. These indicators explain 3.575% and 2.177% of the variation for urban and rural areas, respectively (Supplementary Table S3 and S4). It is consistent with the literature that race and ethnicity have been observed to contribute to vulnerability (Cutter, Boruff et al. 2003, Burton and Cutter 2008, Schmidtlein, Deutsch et al. 2008, Holand, Lujala et al. 2011, Aksha, Juran et al. 2019; Cutter, Ash et al. 2014, Berke, Yu et al. 2019, Drakes, Tate et al. 2021; Solangaarachchi, Griffin et al. 2012, Wigtil, Hammer et al. 2016, Akinola, Adegoke et al. 2019), potentially due to the unequal access to resources, cultural differences, and the social, economic, and political marginalisation that is often associated with racial disparities.

In Theme 4 of housing characteristics (Fig 2), some common indicators identified in both urban and rural areas include households without cars (α= 0.799 for urban and 0.686 for rural), multi-unit houses (α= 0.779 and 0.746), households without internet (α= 0.619 and 0.809), and mobile living (α= -0.782 and 0.752). The indicator of renters (α= 0.764) contributes to vulnerability only in urban areas, possibly due to the fact that renters are more likely to live in urban areas with more available rental houses and easier access to jobs compared to rural areas. However, low mortgage (α= 0.750) and diversity of housing (α= 0.643) contribute to vulnerability only in rural areas where housing prices are relatively lower and more mixed land use are observed compared to urban areas with strict zoning systems. In additional, mobile living houses function differently in urban and rural areas. Urban areas with less mobile living houses (α= -0.782; i.e., caravan parks and manufactured houses) tend to be more vulnerable, possibly due to that such areas with houses less flexible to move would be subject to more loss of assets during natural hazards; while rural areas with more mobile living houses (α= 0.752) tend to be more vulnerable, possibly due to that mobile living facilities (e.g., camping sites, caravan parks, and movable cabins) are more common in rural space. These indicators explain 7.167% and 6.096% of the variation for urban and rural areas, respectively (Supplementary Table S3 and S4).

In Theme 5 of built environment (Fig 2), some common indicators are identified in both urban and rural areas, including the distance to commercial places (α= 0.841 for urban and 0.859 for rural), public service (α= 0.728 and 0.832), public transit (α= 0.782 and 0.676), and the nearest cities (α= 0.796 and 0.818), density of roads and railways (α= 0.999 and 0.974), connectivity

design (α= 0.999 and 0.969), and density of buildings (α= 0.999 and 0.929). It means that urban and rural areas with higher density of buildings, roads and railways, correspondingly more traffic connectivity (e.g., crossing, roundabout, motorway junctions, slipway, stop signs, traffic signals, and turning circles) tend to have higher levels of vulnerability. It is aligning with the literature concluding that the degree of development of the built environment is associated with the expected structural losses from hazard events (Burton and Cutter 2008, de Oliveira Mendes 2009, Krishnan, Ananthan et al. 2019; Cutter, Boruff et al. 2003, Holand, Lujala et al. 2011, Chen, Cutter et al. 2013; Cutter, Ash et al. 2014, Kirby, Reams et al. 2019, Cumberbatch, Drakes et al. 2020). Furthermore, urban and rural areas with longer distances to public facilities (e.g., commercial places, public transit and service) and the nearest city may present higher levels of vulnerability given people living there may be more difficulty to access social resources and support in face of natural disasters. Moreover, street design (α= 0.886, e.g., the configuration of streetlamps and benches) contributes to vulnerability only in urban areas, possibly due to that the pedestrian-friendly neighbourhoods are more common in urban areas compared to more car-dependent environment in rural areas. These indicators explain 8.462% and 7.397% of the variation for urban and rural areas, respectively (Supplementary Table S3 and S4).

Table S3. Results of the principal component analysis and the loading factors of 11 principal components in urban areas

	PC 1	PC 2	PC 3	PC 4	PC 5	PC 6	PC 7	PC 8	PC 9	PC 10	Communalities
No car	**0.799**	-0.038	0.168	-0.115	0.048	0.018	0.243	0.032	0.002	-0.030	0.745
Multi-unit houses	**0.779**	-0.106	0.124	-0.148	-0.264	0.002	0.141	-0.016	0.140	-0.031	0.765
Renters	**0.764**	-0.176	0.111	-0.138	0.148	0.001	0.143	0.147	0.189	-0.030	0.746
Age above 65	0.021	**0.942**	-0.107	0.019	-0.103	-0.002	0.092	-0.007	-0.002	0.015	0.918
Median age	0.075	**0.924**	-0.139	0.049	-0.168	-0.003	0.022	-0.032	-0.047	0.022	0.914
Not labour force	0.110	**0.806**	0.193	0.012	0.274	-0.001	0.061	0.021	-0.054	0.002	0.781
Need assistance	0.058	**0.706**	0.057	-0.025	0.247	-0.003	0.081	0.075	0.109	0.005	0.591
Non-English speaker	0.159	-0.081	**0.909**	-0.159	0.120	-0.002	0.014	0.001	0.021	-0.036	0.899
Minority	0.238	-0.046	**0.859**	-0.172	-0.027	0.002	-0.064	-0.099	-0.028	-0.024	0.843
Bad English	0.145	0.052	**0.835**	-0.069	0.144	-0.003	0.076	0.026	0.042	-0.035	0.757
Distance to commercial	-0.138	-0.019	-0.104	**0.841**	0.032	-0.001	0.010	0.079	-0.015	0.020	0.745
Distance to public transits	-0.055	0.005	-0.059	**0.782**	0.009	-0.001	0.005	0.002	0.001	0.008	0.618
Distance to public service	-0.218	-0.053	-0.130	**0.728**	0.117	-0.001	0.054	-0.042	-0.005	0.014	0.616
Distance to healthcare	-0.121	0.045	-0.174	**0.715**	0.154	-0.001	0.020	-0.083	-0.025	-0.008	0.59
Non-MPA occupation	-0.129	0.010	0.081	0.148	**0.859**	-0.001	0.080	0.058	-0.015	-0.018	0.794
Low education	-0.274	0.272	-0.123	0.191	**0.761**	0.001	0.071	0.086	-0.050	-0.005	0.795
Connectivity design	0.010	-0.003	0.001	-0.001	0.001	**0.999**	-0.001	0.001	0.001	-0.002	0.999
Density of buildings	0.010	-0.003	0.001	-0.002	0.001	**0.999**	-0.001	0.001	0.001	-0.002	0.999
Density of roads and railways	0.009	-0.003	0.001	-0.001	0.001	**0.999**	-0.001	-0.001	0.001	-0.002	0.999
Retired village	0.097	0.153	-0.059	0.021	-0.055	0.001	**0.616**	-0.127	-0.148	-0.008	0.458
Distance to cities	-0.061	0.045	-0.080	0.058	0.026	0.001	-0.046	**0.796**	-0.081	0.009	0.659
Mobile living	-0.009	0.017	-0.042	-0.022	0.008	0.001	0.183	0.035	**-0.782**	0.027	0.649
Street design	0.001	-0.035	0.033	-0.029	0.018	0.003	0.110	-0.038	0.087	**0.886**	0.809
Rotated Squared Loadings	4.452	4.125	3.575	3.177	3.12	2.999	1.639	1.21	1.076	1.021	
% of Variance	11.715	10.855	9.407	8.36	8.211	7.892	4.313	3.185	2.831	2.686	
Cumulative %	11.715	22.57	31.977	40.337	48.547	56.439	60.752	63.937	66.767	69.454	
KMO of Sampling Adequacy	0.794										
Bartlett's Test of Sphericity	1299179										

Note: PC=principal components; KMO=Kaiser-Meyer-Olkin measures

Bold values indicate the loading factors of variables that significantly contribute to a certain PC

Table S4. Results of the principal component analysis and the loading factors of 11 principal components in rural areas

	PC 1	PC 2	PC 3	PC 4	PC 5	PC 6	PC 7	PC 8	PC 9	PC 10	PC 11	Communalities
Median age	**0.932**	-0.042	0.001	0.015	-0.029	-0.027	-0.109	-0.162	0.082	-0.004	0.024	0.918
Age above 65	**0.918**	-0.072	-0.002	0.067	-0.027	-0.021	-0.063	-0.102	0.214	0.02	0.002	0.914
Not labour force	**0.817**	-0.058	0.003	0.157	0.015	0.044	0.198	0.122	0.138	0.063	-0.018	0.775
Age 0-18	**0.798**	-0.008	0.003	0.039	-0.192	-0.131	0.054	0.188	0.22	-0.006	-0.051	0.781
Need child care	**0.699**	0.01	-0.008	-0.05	-0.239	-0.066	-0.109	0.047	0.351	-0.148	-0.029	0.712
Distance to commercial	-0.039	**0.859**	0.001	0.017	-0.056	-0.057	-0.037	0.053	-0.07	0.005	0.002	0.755
Distance to public service	-0.038	**0.832**	0.001	-0.004	-0.026	-0.031	-0.020	0.086	-0.024	0.024	-0.017	0.705
Distance to cities	-0.085	**0.818**	-0.002	-0.01	-0.026	-0.012	-0.023	0.132	0.030	-0.02	0.015	0.698
Distance to public transits	-0.046	**0.676**	-0.001	-0.009	-0.005	-0.099	0.018	0.223	-0.018	0.225	-0.043	0.572
Density of roads and railways	0.002	0.001	**0.974**	-0.001	-0.005	-0.001	-0.004	-0.001	0.002	-0.004	-0.002	0.948
Connectivity design	0.001	-0.001	**0.969**	-0.001	-0.002	0.001	-0.004	-0.002	0.004	-0.004	-0.002	0.940
Density of buildings	-0.002	-0.002	**0.929**	-0.009	0.001	0.003	0.004	0.002	-0.01	0.005	-0.006	0.864
No internet	0.162	0.093	-0.005	**0.809**	0.098	-0.125	0.172	0.122	-0.044	0.168	0.011	0.790
Low mortgage	0.22	-0.113	-0.009	**0.75**	-0.026	-0.032	0.222	-0.045	0.068	-0.219	0.012	0.729
No car	0.017	0.098	-0.002	**0.686**	0.389	0.07	0.050	0.352	0.028	0.115	-0.019	0.778
Multi-unit houses	0.001	-0.078	-0.002	0.229	**0.746**	0.247	-0.088	-0.178	0.080	-0.007	-0.059	0.725
Diversity of housing	0.147	-0.031	-0.002	-0.084	**0.643**	0.043	0.104	0.030	0.004	0.037	0.098	0.468
Minority	0.114	-0.137	0.008	-0.133	0.203	**0.813**	-0.083	-0.034	-0.017	-0.048	0.003	0.761
Ethnic diversity	0.039	-0.072	0.004	-0.072	0.089	**0.687**	-0.063	-0.081	0.031	-0.016	-0.023	0.505
Bad English	-0.002	-0.091	-0.007	0.161	-0.031	**0.668**	0.154	0.093	-0.093	-0.005	0.023	0.523
Non-MPA occupation	0.017	-0.133	0.003	0.144	-0.016	-0.005	**0.774**	-0.029	-0.041	0.061	-0.068	0.648
Low education	0.327	0.053	-0.005	0.170	-0.16	-0.179	**0.678**	-0.18	0.046	-0.022	-0.005	0.691
Unemployed	-0.136	0.031	0.001	0.011	0.266	0.123	**0.609**	0.219	0.046	-0.011	0.009	0.527
Indigenous	-0.161	0.363	-0.001	0.113	0.144	-0.108	0.191	**0.671**	0.105	0.272	-0.061	0.778
Multi-family HH	-0.066	0.126	-0.002	0.219	-0.148	0.070	-0.087	**0.638**	-0.042	-0.229	0.058	0.567
Female	0.183	-0.254	-0.002	0.008	0.165	-0.020	0.055	0.097	**0.763**	-0.116	0.004	0.735
Mobile living	0.025	0.007	-0.005	-0.028	-0.076	-0.002	0.045	0.006	-0.055	**0.752**	0.054	0.582
Street design	-0.088	0.044	0.007	-0.008	0.026	0.052	0.084	-0.088	0.185	0.072	**0.793**	0.696
Rotated Squared Loadings	4.743	3.604	2.753	2.426	2.283	2.177	2.087	2.005	1.533	1.387	1.04	
% of Variance	12.482	9.483	7.244	6.385	6.007	5.73	5.492	5.277	4.033	3.65	2.738	
Cumulative %	12.482	21.965	29.208	35.593	41.601	47.331	52.823	58.1	62.133	65.783	68.521	
KMO of Sampling Adequacy	0.797											
Bartlett's Test of Sphericity	422744.8											

Note: PC=principal components; KMO=Kaiser-Meyer-Olkin measures
Bold values indicate the loading factors of variables that significantly contribute to a certain PC

Table S5. Reclassification of 10 principal components of the vulnerability index in urban areas in five themes

**Theme**	**Principal component (PC)**	**Indicator**	**Loadings**
**Theme 1: Socioeconomic status**	PC 5 (Socioeconomic)	Non-MPA occupation	0.859
		Low education	0.761
**Theme 2: Demographics & disability**	PC 2 (Demographic—Age)	Age above 65	0.942
		Median age	0.924
		Not labour force	0.806
		Need assistance	0.706
**Theme 3: Minority & languages**	PC 3 (Minority & language)	Non-English speaker	0.909
		Minority	0.859
		Bad English	0.835
**Theme 4: Housing characteristics**	PC 1 (Housing and transportation)	No car	0.799
		Multi-unit houses	0.779
		Renters	0.764
	PC 7 (Housing)	No internet	0.619
	PC 9 (Housing)	Mobile living	-0.782
**Theme 5: Built environment**	PC 4 (Built environment—Distance/accessibility)	Distance to commercial	0.841
		Distance to public transits	0.782
		Distance to public service	0.728
		Distance to healthcare	0.715
	PC 6 (Built environment—Density and design)	Connectivity design	0.999
		Density of buildings	0.999
		Density of roads and railways	0.999
	PC 8 (Built environment—Distance/accessibility)	Distance to cities	0.796
	PC 10 (Built environment—Design)	Street design	0.886

Table S6. Reclassification of 11 principal components of the vulnerability index in rural areas in five themes

Theme	Principal component (PC)	Indicator	Loadings
**Theme 1: Socioeconomic status**	PC 7 (Socioeconomic)	Non-MPA occupation	0.774
		Low education	0.678
		Unemployed	0.609
**Theme 2: Demographics & disability**	PC 1 (Demographic—Age)	Median age	0.932
		Age above 65	0.918
		Not labour force	0.817
		Age 0-18	0.798
		Need child care	0.699
	PC 8 (Demographic)	Indigenous	0.671
		Multi-family HH	0.638
	PC 9 (Demographic)	Female	0.763
**Theme 3: Minority & languages**	PC 6 (Minority & language)	Minority	0.813
		Ethnic diversity	0.687
		Bad English	0.668
**Theme 4: Housing characteristics**	PC 4 (Housing and transportation)	No internet	0.809
		Low mortgage	0.75
		No car	0.686
	PC 5 (Housing)	Multi-unit houses	0.746
		Diversity of housing	0.643
	PC 10 (Housing)	Mobile living	0.752
**Theme 5: Built environment**	PC 2 (Built environment—Distance/accessibility)	Distance to commercial	0.859
		Distance to public service	0.832
		Distance to cities	0.818
		Distance to public transits	0.676
	PC 3 (Built environment—Density and design)	Density of roads and railways	0.974
		Connectivity design	0.969
		Density of buildings	0.929
	PC 11 (Built environment—Design)	Street design	0.793

Note 3 (Details of the most vulnerable areas in capital cities)

For Theme 1 (Supplementary Fig S2), the most vulnerable areas in terms of socioeconomic status appear in the west (e.g., Windsor - Bligh Park, St Marys - North St Marys), the southwest (e.g., Ashcroft - Busby – Miller, Bonnyrigg Heights – Bonnyrigg), and the south of Sydney (e.g., Campbelltown – Woodbine, Bradbury – Wedderburn); in the north (e.g., Burwood) and southeast Melbourne (e.g., Clayton, Doveton, Cranbourne); in the south and southwest of Brisbane (e.g., Logan, Springwood – Kingston, Beenleigh, Inala – Richlands, Ipswich); in the northeast (e.g., Elizabeth, Paralowie) and the south (e.g., Hackham West, Huntfield Heights) of Adelaide; in the south and northeast of Perth (e.g., Mandurah, Canning Vale – East, Bentley - Wilson - St James, Midland – Guildford); in the western bay of Hobart (e.g., Moonah, Glenorchy) and the eastern bay of Hobart (e.g., Bridgewater – Gagebrook, Risdon Vale, Mornington – Warrane); in the urban fringe of Darwin (e.g., Howard Springs, Berrimah); and in several small suburbs in Australian Capital Territory (e.g., Canberra East, Acton, Bruce).

For Theme 2 (Supplementary Fig S3), the most vulnerable areas in terms of demographics and disability have less obvious patterns that can be generalised but more spread out sporadically across entire urban space outside of inner cities in Sydney, Brisbane, Adelaide, Perth, and ACT. The concentrations of such vulnerable areas are more obvious to see in the southeast coast of Melbourne (e.g., Flinders, Mornington, Mount Martha, Dromana, Point Nepean), in the west of Hobart (e.g., Taroona - Bonnet Hill, New Town, Glenorchy, Claremont) and in Darwin (e.g., Lyons).

For Theme 3 (Supplementary Fig S4), the most vulnerable areas in terms of minority and languages appear in the west and south of Sydney (e.g., Liverpool, Blacktown); in the southwest of Melbourne (e.g., Springvale, Dandenong), the north (e.g., Thomastown, Campbellfield – Coolaroo, Roxburgh Park – Somerton, Craigieburn) and the west of Melbourne (e.g., Thomastown, Campbellfield – Coolaroo, Roxburgh Park – Somerton, Craigieburn); in the south and southwest of Brisbane (e.g., Sunnybank, Sunnybank Hills, Calamvale – Stretton); in Adelaide (e.g., Parafield), Perth (e.g., Jandakot) and Darwin (e.g., Humpty Doo).

For Theme 4 (Supplementary Fig S5), the most vulnerable areas in terms of housing characteristics appear in a few suburbs in Sydney (e.g., Bilpin - Colo - St Albans); in the northwest of Melbourne (e.g., Sunbury – South, Gisborne); in the northwest of Brisbane (e.g., Esk in Ipswich Hinterland) and the south of Brisbane (e.g., Boonah and Beaudesert); in the north corner of Adelaide (e.g., Virginia – Waterloo) and the east of Adelaide (e.g., Mount Barker); in the north of Perth (e.g., Tapping - Ashby – Sinagra) and in the south of Perth (e.g., Mandurah – North, Halls Head – Erskine, Pinjarra); in Hobart (e.g., Bridgewater – Gagebrook, Glenorchy, South Hobart - Fern Tree, Sorell – Richmond); in Darwin (e.g., Weddell); in the southwest of ACT (e.g., Aranda, Ainslie, Campbell, Calwell).

For Theme 5 (Supplementary Fig S6), the most vulnerable areas in terms of built environment in capital cities share some common patterns — largely appearing close to and/or on the urban fringe, far away from the inner cities.

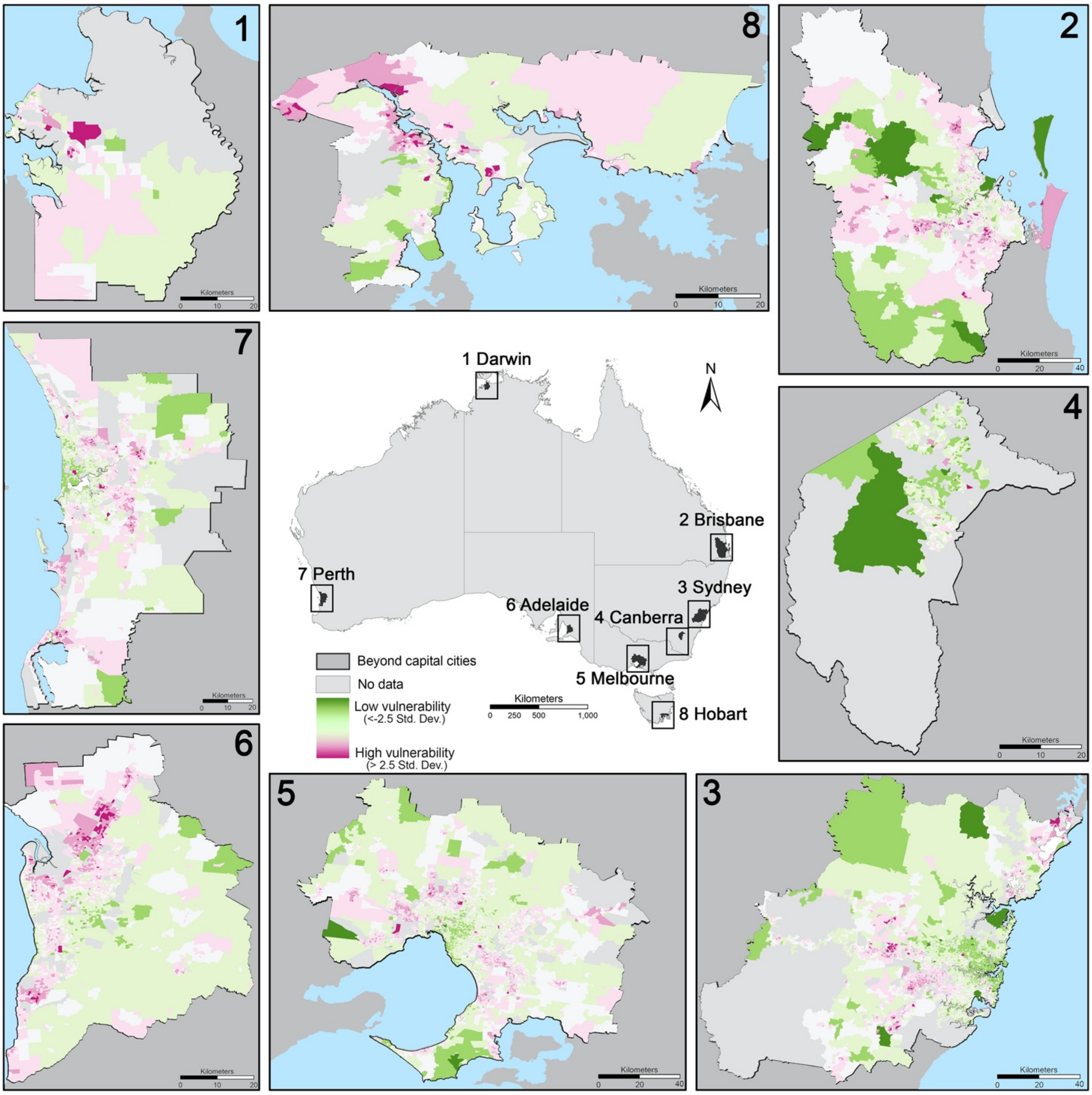

Figure S2. Spatial patterns of the vulnerability index in Theme 1 (socioeconomic status) in urban areas of eight capital cities

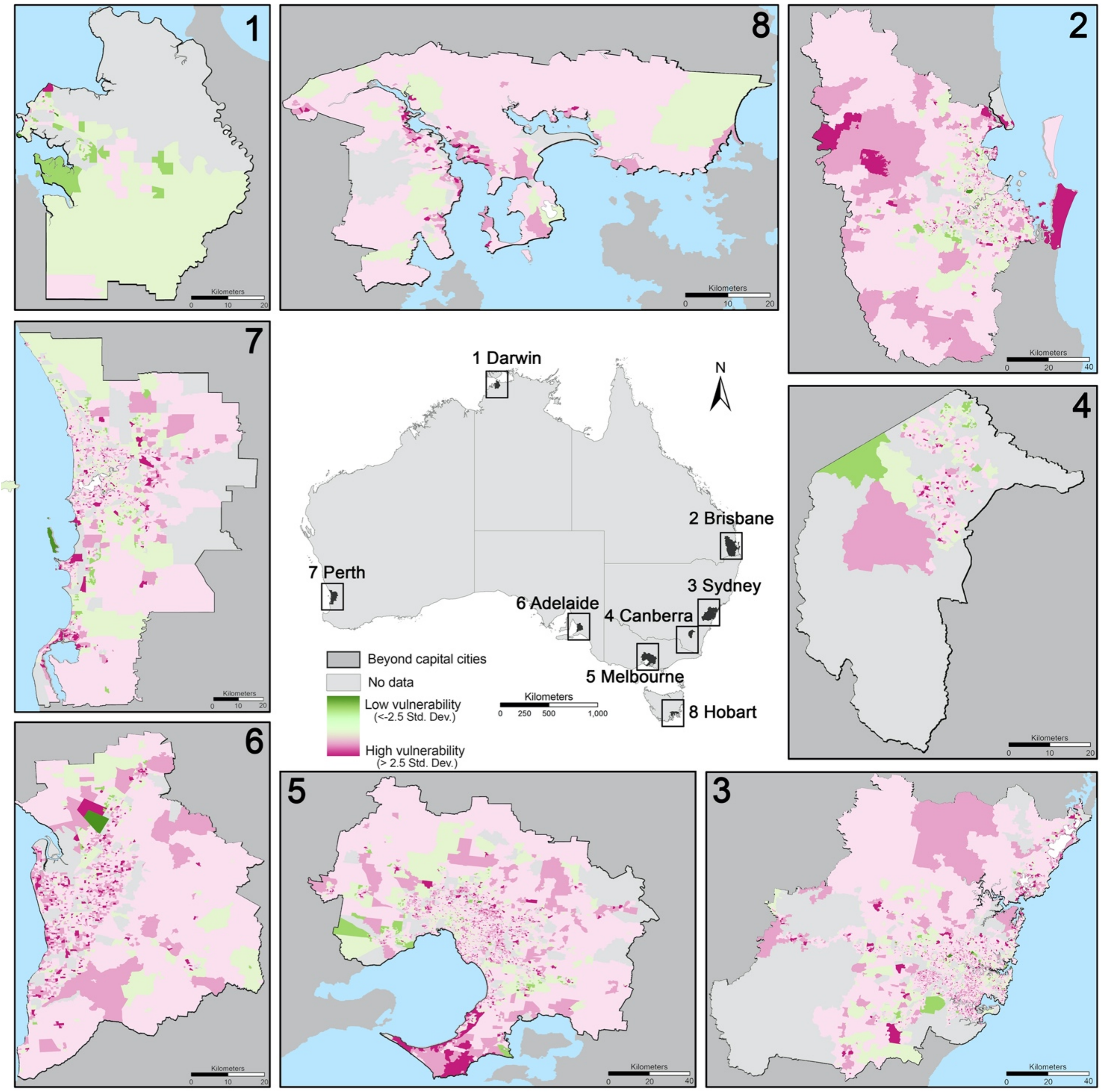

Figure S3. Spatial patterns of the vulnerability index in Theme 2 (demographics & disability) in urban areas of eight capital cities

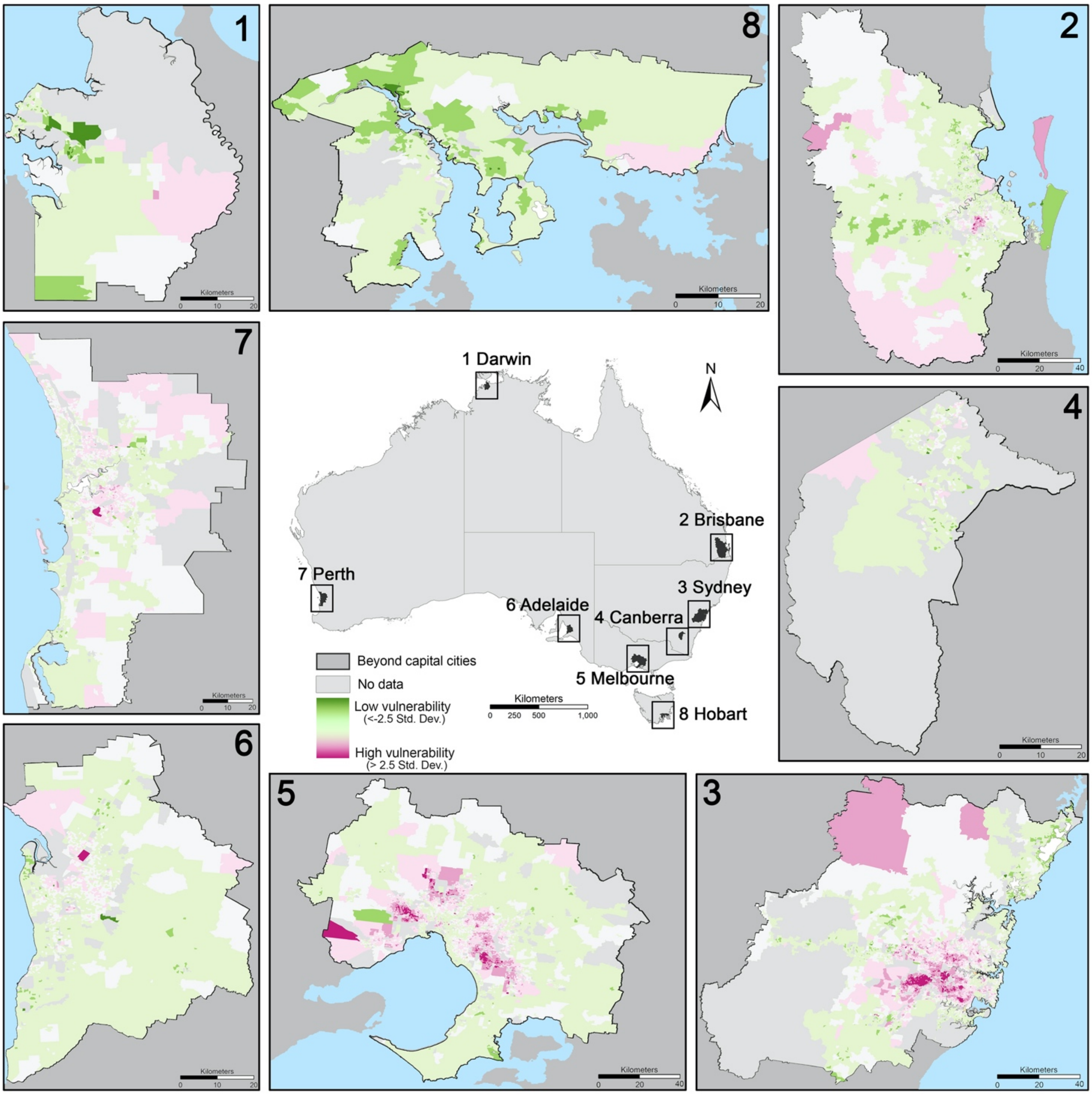

Figure S4. Spatial patterns of the vulnerability index in Theme 3 (minority & languages) in urban areas of eight capital cities

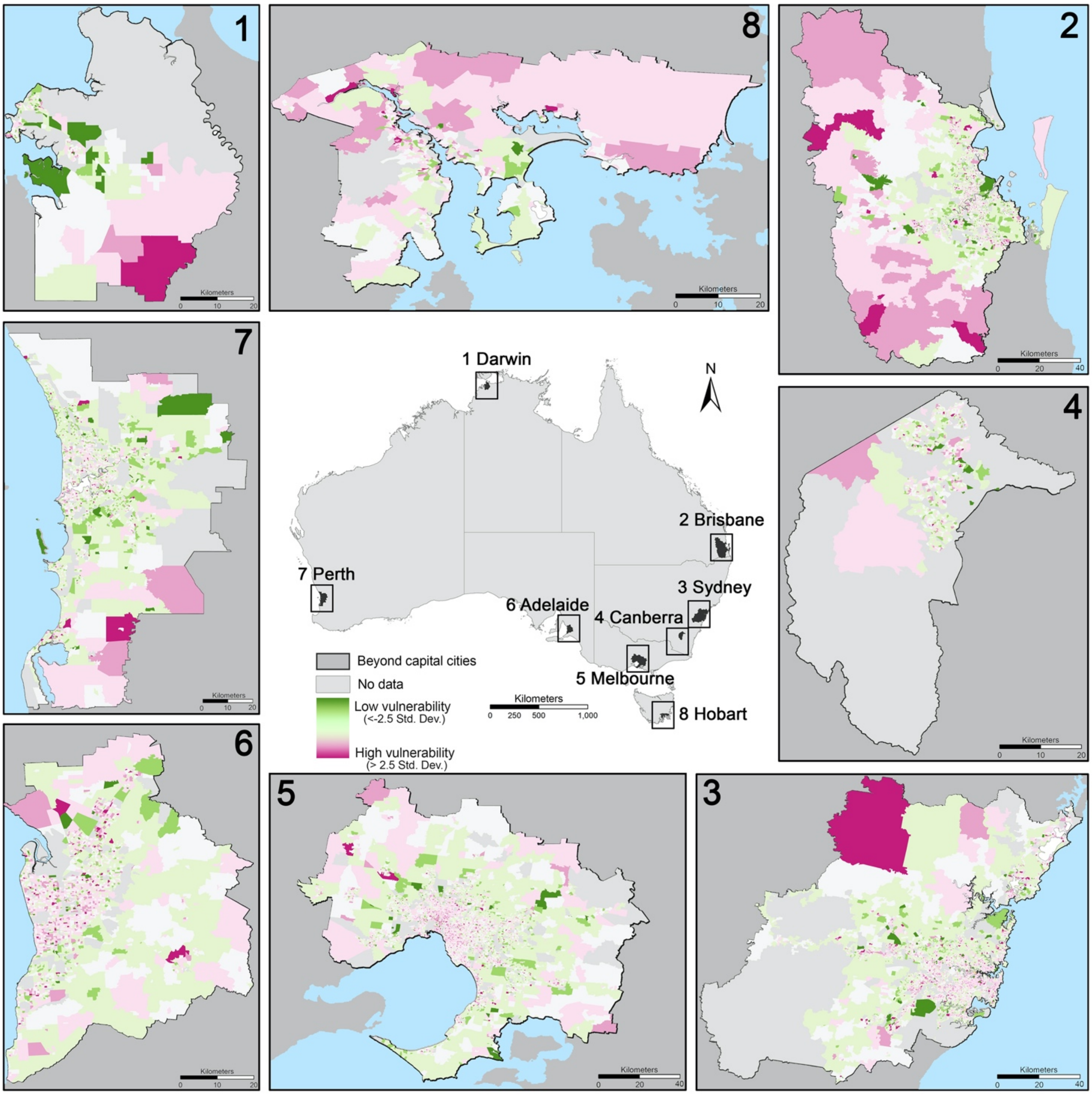

Figure S5. Spatial patterns of the vulnerability index in Theme 4 (housing characteristics) in urban areas of eight capital cities

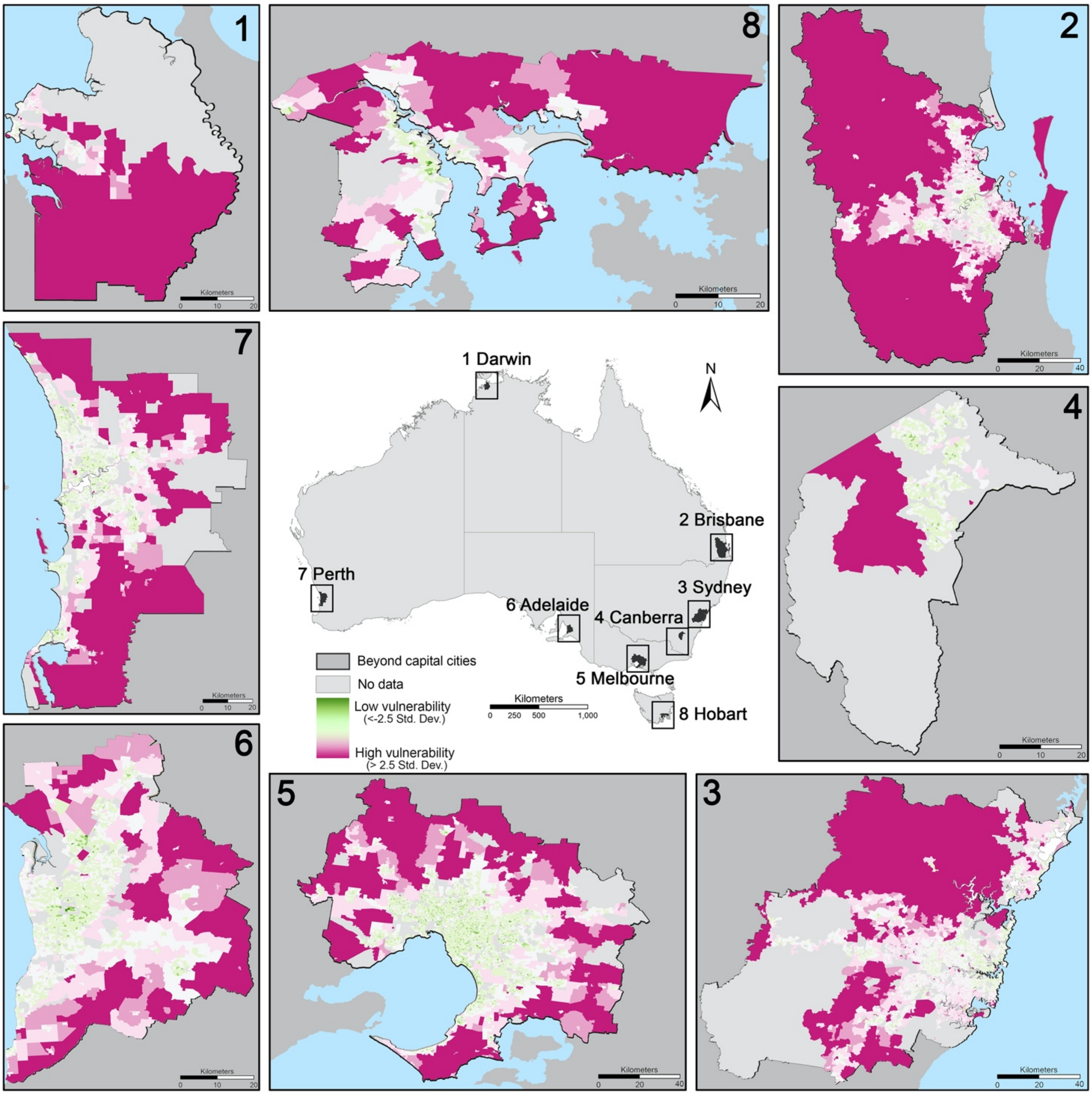

Figure S6. Spatial patterns of the vulnerability index in Theme 5 (built environment) in urban areas of eight capital cities

Table S7. The most vulnerable areas in rural and urban areas in different themes

	Rural areas	Urban areas
Overall	West Australia: Exmouth, Leinster, Meekatharra, East Pilbara, Roebuck, Kimberley, Kununurra<br>Northern Territory: Alice Springs, Barkly, Katherine, Daly-Tiwi West Arnhem (south of Darwin)<br>South Australia: Outback	Sydney: North (Bilpin - Colo - St Albans); Southwest: (The Oaks – Oakdale, Rosemeadow - Glen Alpine, Cobbitty – Leppington, Austral – Greendale, Bradbury – Wedderburn)<br>Melbourne: Wyndham Vale, Melton, Romsey, Wallan, Koo Wee Rup<br>Brisbane: Logan, Loganlea, Springwood – Kingston, Beenleigh, Inala – Richlands, Ipswich – Central<br>Adelaide: Virginia - Waterloo Corner, Adelaide Hills, Mount Barker Region<br>Perth: North (Carabooda – Pinjar, Bullsbrook, Chidlow, Gidgegannup); East (Lesmurdie - Bickley – Carmel, Serpentine – Jarrahdale); South (Pinjarra, Dawesville – Bouvard)<br>Hobart: Bridgewater – Gagebrook, New Norfolk, Brighton – Pontville, Sorell – Richmond, Risdon Vale, Dodges Ferry – Lewisham<br>Darwin: South (Weddell, Howard Springs)<br>ACT: ACT - South West, Page
Theme 1	West Australia: Exmouth, Leinster, Meekatharra, East Pilbara, Roebuck, Kimberley, Kununurra<br>Northern Territory: Alice Springs, Barkly, Katherine, Daly-Tiwi West Arnhem (south of Darwin)<br>South Australia: Outback	Sydney: West (Windsor - Bligh Park, Lethbridge Park – Tregear, St Marys - North St Marys, Doonside – Woodcroft); Southwest (Ashcroft - Busby – Miller, Bonnyrigg Heights – Bonnyrigg, Macquarie Fields – Glenfield); South (Claymore - Eagle Vale – Raby, Campbelltown – Woodbine, Bradbury – Wedderburn)<br>Melbourne: Laverton, Broadmeadows, Campbellfield – Coolaroo, Kingsbury, Bundoora – North, Burwood, Southeast (Clayton, Doveton, Cranbourne, Frankston North, Seaford)<br>Brisbane: Logan, Loganlea, Springwood – Kingston, Beenleigh, Inala – Richlands, Ipswich – Central<br>Adelaide: Northeast (Elizabeth, Paralowie, Enfield - Blair Athol); South (Hackham West - Huntfield Heights, Christie Downs)<br>Perth: South (Mandurah), Canning Vale – East, Bentley - Wilson - St James, Midland – Guildford<br>Hobart: West bay (Moonah, Glenorchy, Mount Nelson – Dynnyrne); East bay (Bridgewater – Gagebrook, Risdon Vale, Mornington – Warrane, Hobart - North East)<br>Darwin: Howard Springs, Berrimah<br>ACT: Canberra East, Acton, Bruce
Theme 2	West Australia: Derby-West Kimberley<br>Northern Territory: North (West Arnhem, Barkey, Yuendumu Anmatjere)	Sydney, Brisbane, Adelaide: more sporadically spread out in suburbs rather than inner city<br>Melbourne: Southeast coast (Flinders, Mornington, Mount Martha, Dromana, Point Nepean)<br>Perth: spreading out<br>Hobart: West (Taroona - Bonnet Hill, New Town, Glenorchy, Claremont); East: Bellerive – Rosny, Mornington – Warrane,<br>Darwin: Lyons<br>ACT: spreading out

Theme 3	West Australia: Midwest (Meekatharra, East Pilbara)	Sydney: Liverpool, Blacktown<br>Melbourne: Southwest; Springvale, Dandenong; North (Thomastown, Campbellfield – Coolaroo, Roxburgh Park – Somerton, Craigieburn); West: Sunshine North, Braybrook, St Albans – South, Kings Park, Wyndham Vale<br>Brisbane: Ipswich Hinterland, Sunnybank, Sunnybank Hills, Calamvale – Stretton<br>Adelaide: Parafield<br>Perth: Jandakot<br>Darwin: Humpty Doo
Theme 4	West Australia: (East Pilbara, Halls Creek Derby-West Kimberley, Hall Creek)<br>Northern Territory: Tanami, Sandover-Plenty, Barkly, Daly<br>South Australia: West Coast, outback<br>Queensland: Central highlands	Sydney: Bilpin - Colo - St Albans<br>Melbourne: Northwest: Sunbury – South, Gisborne<br>Brisbane: Northwest (Esk in Ipswich Hinterland); South: Boonah and Beaudesert<br>Adelaide: more sporadically spread out in suburbs plus Virginia - Waterloo Corner in the north and Mount Barker in the east<br>Perth: North (Tapping - Ashby – Sinagra); South (Mandurah – North, Halls Head – Erskine, Pinjarra)<br>Hobart: Bridgewater – Gagebrook, Glenorchy, South Hobart - Fern Tree, Sorell – Richmond<br>Darwin: Weddell<br>ACT: ACT - South West, Aranda, Ainslie, Campbell, Calwell
Theme 5	West Australia: Roebuck (coastal), East Pilbara, Meekatharra, Exmouth, Kambalda, Coolgardie<br>Northern Territory: Tanami, Sandover – plenty<br>Queensland: Far central west	Sydney, Melbourne, Brisbane, Perth: Areas on the urban fringe and outer rings of urban space<br>Adelaide: Adelaide Hills, Mount Barker Region, Hahndorf – Echunga, Clarendon<br>ACT: ACT - South West

Note: Theme 1: socioeconomic status; Theme 2: demographic composition & disability; Theme 3: minority status & language; Theme 4: housing type & transportation; Theme 5: built environment

Table S8. Statistic summary of the vulnerability index in urban and rural areas

	**New South Wales**		**Victoria**		**Queensland**		**South Australia**		**West Australia**		**Tasmania**		**North Territory**		**Australian Capital Territory**
	**Urban**	**Rural**	**Urban**	**Rural**	**Urban**	**Rural**	**Urban**	**Rural**	**Urban**	**Rural**	**Urban**	**Rural**	**Urban**	**Rural**	**Urban**
Mean	0.897	-0.033	-0.491	-0.397	0.102	-0.152	0.122	0.186	-0.851	0.452	0.756	-0.171	-0.624	7.041	-2.446
Std. Dev	3.013	3.076	2.410	2.382	3.082	2.879	2.463	2.813	2.290	4.318	3.211	1.959	4.019	7.467	2.173
Min	-14.038	-10.805	-15.260	-9.885	-20.273	-10.702	-30.417	-21.987	-16.372	-12.823	-11.488	-5.248	-18.063	-4.836	-19.224
Max	33.238	59.575	51.240	17.118	33.962	69.094	12.997	23.522	15.114	48.496	16.513	13.223	19.465	25.328	11.572
Number	10741	6911	9980	3674	5179	5943	3039	1036	4306	1411	562	815	308	254	1026

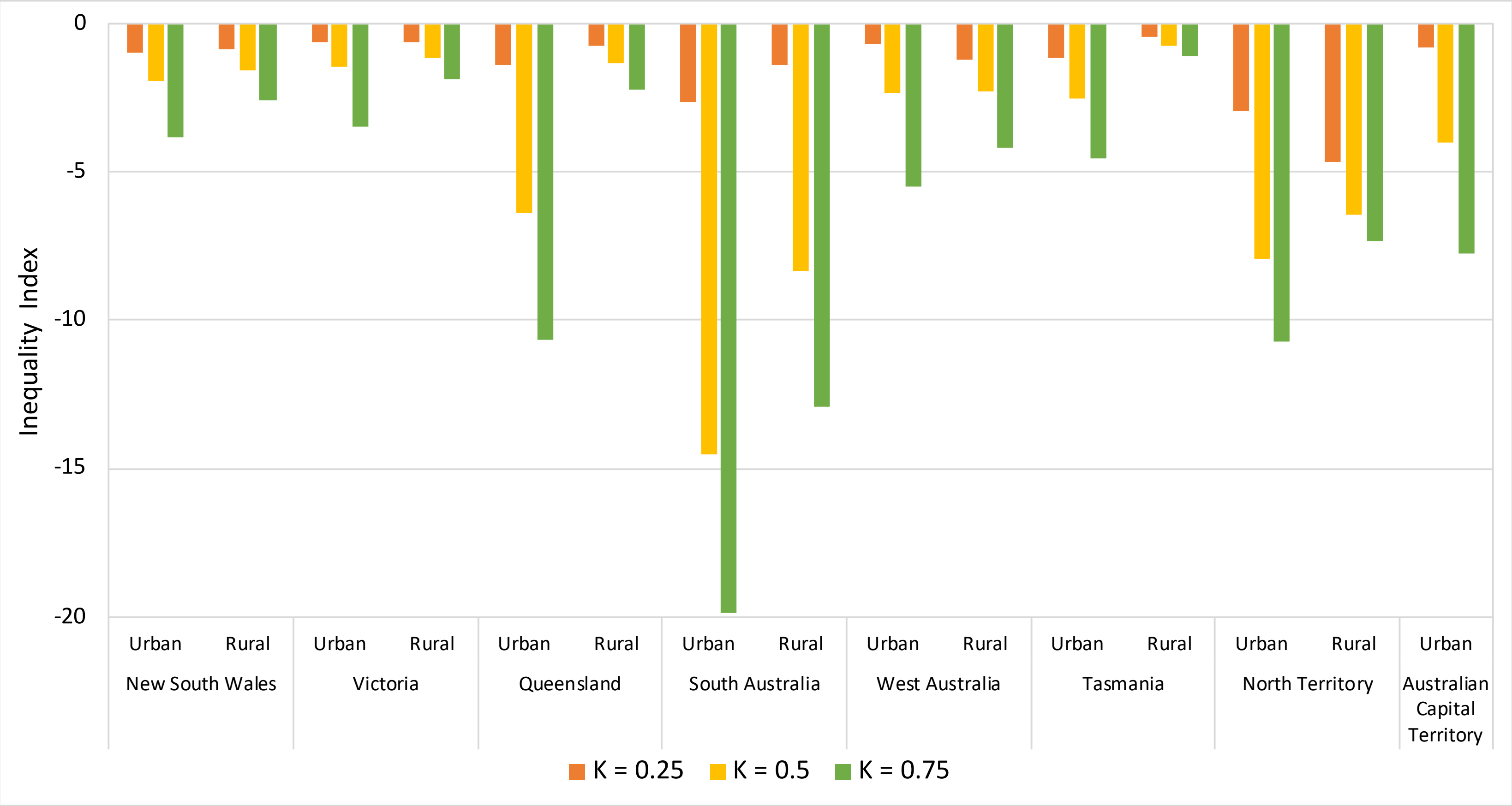

Figure S7. Inequality index of vulnerability in urban and rural areas with different settings of K values (see Methods and Supplementary Table S8). The increase of K values from 0.25 to 0.75 is associated with the increasing magnitude of the inequality index (Supplementary Table S8).

Table S9. Inequity of the vulnerability index in urban and rural areas

	**New South Wales**		**Victoria**		**Queensland**		**South Australia**		**Western Australia**		**Tasmania**		**Northern Territory**		**Australian Capital Territory**
	**Urban**	**Rural**	**Urban**	**Rural**	**Urban**	**Rural**	**Urban**	**Rural**	**Urban**	**Rural**	**Urban**	**Rural**	**Urban**	**Rural**	**Urban**
k=0.25	-0.968	-0.865	-0.639	-0.619	-1.419	-0.720	-2.620	-1.421	-0.677	-1.229	-1.139	-0.417	-2.950	-4.667	-0.795
k=0.5	-1.947	-1.601	-1.435	-1.179	-6.397	-1.342	-14.519	-8.346	-2.330	-2.278	-2.544	-0.764	-7.903	-6.415	-3.989
k=0.75	-3.825	-2.565	-3.449	-1.846	-10.673	-2.220	-19.848	-12.916	-5.491	-4.208	-4.543	-1.071	-10.727	-7.317	-7.772

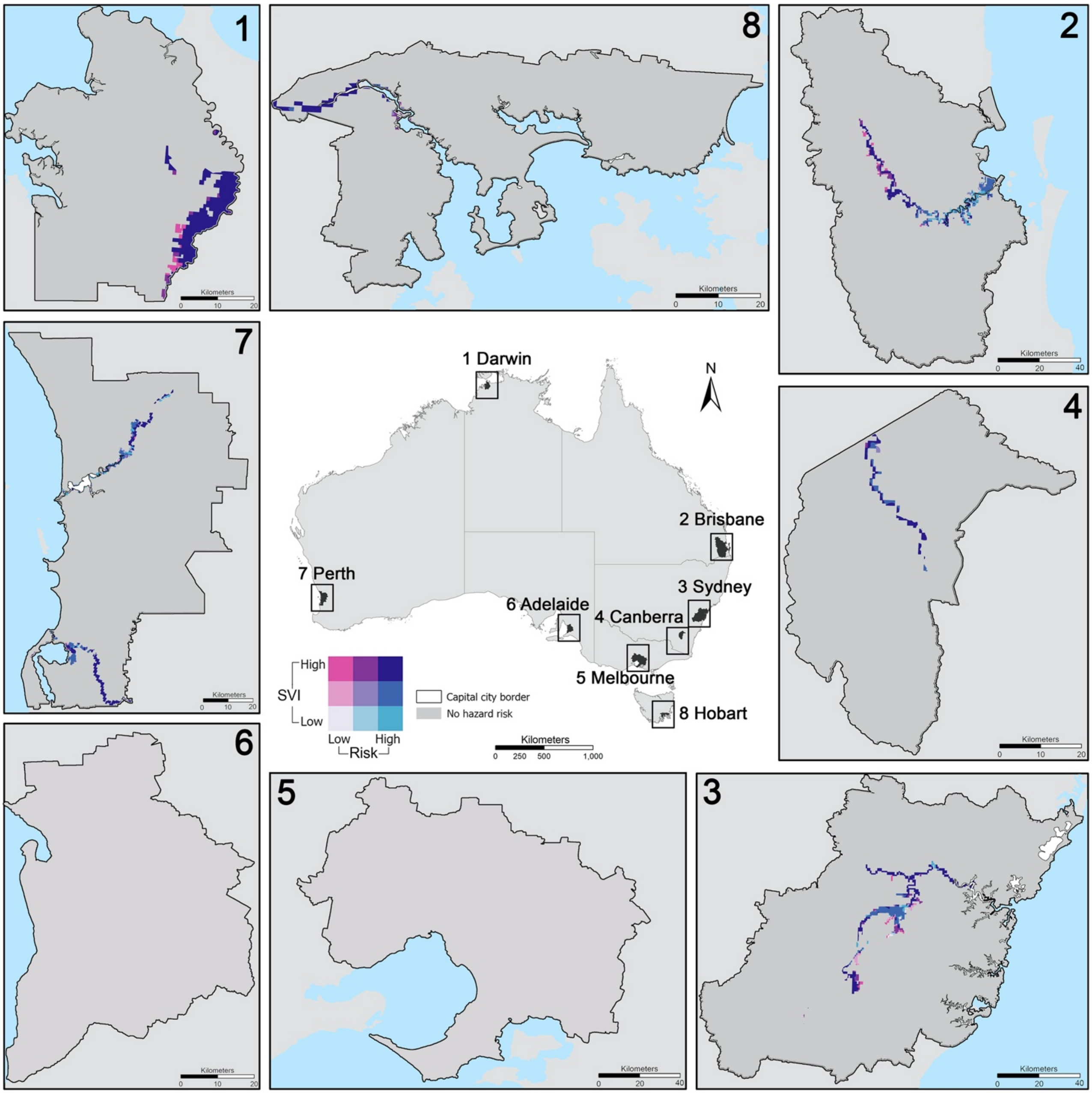

Figure S8. Enlarged capital city maps of the most vulnerable areas highly risky in flood.

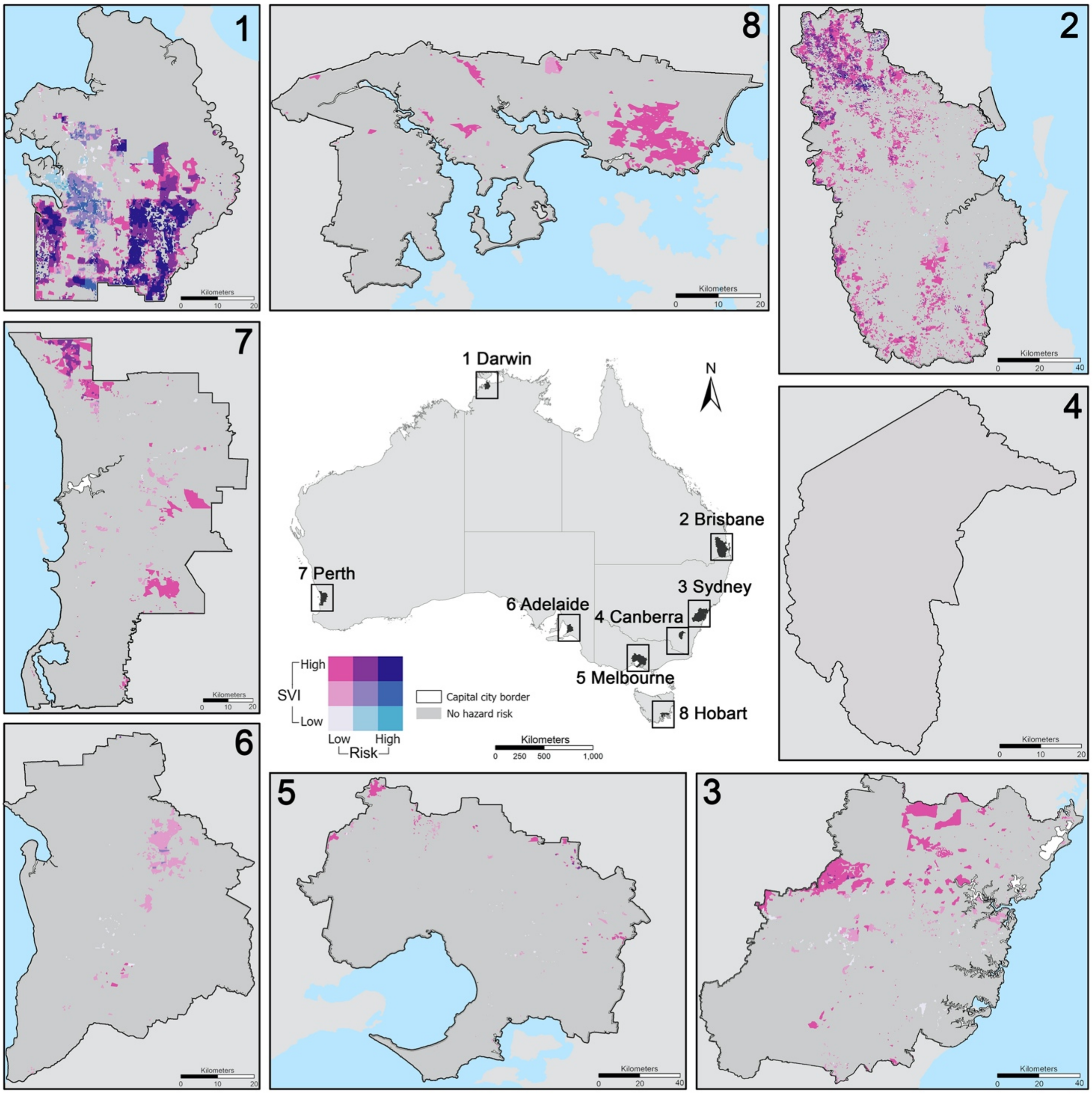

Figure S9. Enlarged capital city maps of the most vulnerable areas highly risky in wildfire. ACT has no hazard-affected areas.

Note 4 (Details of the most vulnerable and highly risky areas in eight capital cities)

We enlarge the maps of capital cities to see more detailed locations of the vulnerable areas highly risky in wildfire and flood (Supplementary Fig S8 and S9). The most vulnerable areas highly risky in flood appear along the hydrological network in capital cities except Adelaide and Melbourne) — along the Brisbane River towards Lake Wivenhoe in Brisbane, along the Parramatta River linking to Lake Burragorang and the outbound ocean harbour in Sydney, along the Murrumbidgee River and Tumut River linking to Eucumbene Dam in ACT, along the Swan River bringing the ocean water toward inland in Perth, along the Adelaide River on the southeasts edge of Darwin and along the River Derwent bringing the ocean water to Meadowbank Dam and Meadowbank Lake (Supplementary Table S9).

The most vulnerable areas highly risky in wildfire appear predominantly in the middle and south of Darwin and the north of Brisbane as the capital city of Northern Territory and Queensland — two topical states in Australia (Supplementary Table S9). The highly vulnerable areas also sporadically appear in the north and northwest of Sydney (e.g., Baulkham Hills and Hawkesbury, Outer West and Blue Mountains), the north and west edge of Melbourne (e.g., Macedon Ranges, Nillumbik – Kinglake), the east of Hobart (Sorell - Dodges Ferry), the northeast of Adelaide (e.g., One Tree Hill, Adelaide Hills) and the north, east and southeast of Perth (e.g., Carabooda – Pinjar, Lesmurdie - Bickley – Carmel, Serpentine - Jarrahdale).

Table S10. The most vulnerable areas highly risky in natural hazards

	**Rural areas**	**Urban areas**
Earthquake	West Australia: Northwest coast (Exmouth, Roebuck,<br>Midwest: East Pilbara<br>Kununurra (border to Northern Territory)<br>Kambalda<br>South Australia: Outback<br>Victoria: Wellington, Yarram, Foster, areas along Western Port Bay	ACT: southwest<br>Melbourne: south coastal areas along the Western Port Bay<br>Adelaide: the major areas in the middle and south
Wildfire	West Australia: Derby<br>Northern Territory: West Arnhem, Kakadu National Park, Daly River<br>Queensland: North peninsula (Cape York)	Brisbane: Caboolture Hinterland, Lockyer Valley – East<br>Sydney - Baulkham Hills and Hawkesbury, Sydney - Outer West and Blue Mountains<br>ACT: Urriarra – Namadgi<br>Melbourne: Macedon Ranges, Nillumbik – Kinglake<br>Hobart: East (Sorell - Dodges Ferry)<br>Adelaide: One Tree Hill, Adelaide Hills<br>Perth: North (Carabooda – Pinjar), East (Lesmurdie - Bickley – Carmel), Southeast (Serpentine - Jarrahdale)
Flood	Queensland: southwestern inland<br>South Australia: northeast corner (outback)	Along the Brisbane River towards Lake Wivenhoe in Brisbane, along the Parramatta River linking to Lake Burragorang and the outbound ocean harbour in Sydney, along the Murrumbidgee River and Tumut River linking to Eucumbene Dam in ACT, along the Swan River bringing the ocean water toward inland in Perth, along the Adelaide River on the southeasts edge of Darwin and along the River Derwent bringing the ocean water to Meadowbank Dam and Meadowbank Lake.

Table S11. Statistic summary of the vulnerability index in earthquake-affected and non-hazard areas

Region	Mean	Std. Dev	Min	Max	Number
Hazard-affected areas					
Sydney	0.90	3.02	-14.04	33.24	10817
Rest of NSW	-0.20	3.14	-10.81	59.57	5421
Melbourne	-0.48	2.41	-15.26	51.24	10130
Rest of Vic.	-0.49	2.36	-9.89	17.12	3575
Brisbane	—	—	—	—	—
Rest of Qld	8.18	7.18	-1.49	20.07	39
Adelaide	0.15	2.47	-30.42	13.00	3133
Rest of SA	0.21	2.89	-9.60	23.52	988
Perth	-0.81	2.33	-16.37	15.11	4420
Rest of WA	0.35	4.00	-12.82	48.50	1573
Hobart	—	—	—	—	—
Rest of Tas.	0.01	2.12	-5.25	13.22	223
Darwin	-0.62	4.02	-18.06	19.47	308
Rest of NT	8.64	6.96	-3.51	22.41	74
Australian Capital Territory	-2.45	2.17	-19.22	11.57	1026
Non-hazard areas					
Sydney	-0.46	2.60	-12.41	12.82	424
Rest of NSW	0.19	2.80	-9.74	15.87	2036
Melbourne	-0.45	2.20	-5.96	8.16	133
Rest of Vic.	-0.08	2.29	-4.92	9.91	409
Brisbane	0.10	3.08	-20.27	33.96	5179
Rest of Qld	-0.17	2.85	-10.70	69.09	5920
Adelaide	0.41	2.03	-3.54	8.77	79
Rest of SA	-0.13	2.69	-21.99	14.45	291
Perth	-0.38	2.70	-16.37	7.84	180
Rest of WA	1.22	5.78	-12.82	38.51	203
Hobart	0.76	3.21	-11.49	16.51	562
Rest of Tas.	-0.23	1.95	-5.25	13.22	664
Darwin	-0.85	3.33	-5.75	7.86	24
Rest of NT	6.58	7.36	-4.84	25.33	219
Australian Capital Territory	-0.46	2.60	-12.41	12.82	424

Note 5 ((Details of the most vulnerable areas highly risky in flood and wildfire)

The mean values and ranges of the vulnerability index in wildfire-affected areas vary substantially across urban and rural areas (Supplementary Figure S10 with the statistical summary provided in Supplementary Table S11). Its mean is highest in Brisbane (13.844), followed by rural West Australia (10.198) and Darwin (7.956) while lowest in rural Victoria (-0.965), followed by rural Tasmania (-0.433) and rural New South Wales (-0.380). In New South Wales, Queensland, Tasmania and Northern Territory, the ranges of the vulnerability index in wildfire-affected urban areas are wider than its ranges in rural areas, while oppositely in Victoria, South Australia, and West Australia.

The mean values and ranges of the vulnerability index in flood-affected areas vary substantially across urban and rural areas (Supplementary Figure S11 with the statistical

summary provided in Supplementary Table S12). Its mean is highest in Darwin as the urban areas of Northern Territory (17.534), followed by rural Northern Territory (9.228) and rural South Australia (7.003) while lowest in rural Victoria (-0.952), followed by rural New South Wales (-0.713) and rural Tasmania (-0.710). The ranges of the vulnerability index in flood-affected areas is widest in rural New South Wales (-10.168 to 51.985), followed by its counterpart Sydney (-11.362 to 29.666) and Brisbane as the urban areas of Queensland (-10.257 to 22.467). Moreover, the ranges of the vulnerability index in flood-affected areas are relatively narrow in Tasmania (-3.651 to 10.197 in urban areas and -4.540 to 4.287 in rural areas) and Darwin as the urban areas in Northern Territory (13.670 to 19.465) compared to other states.

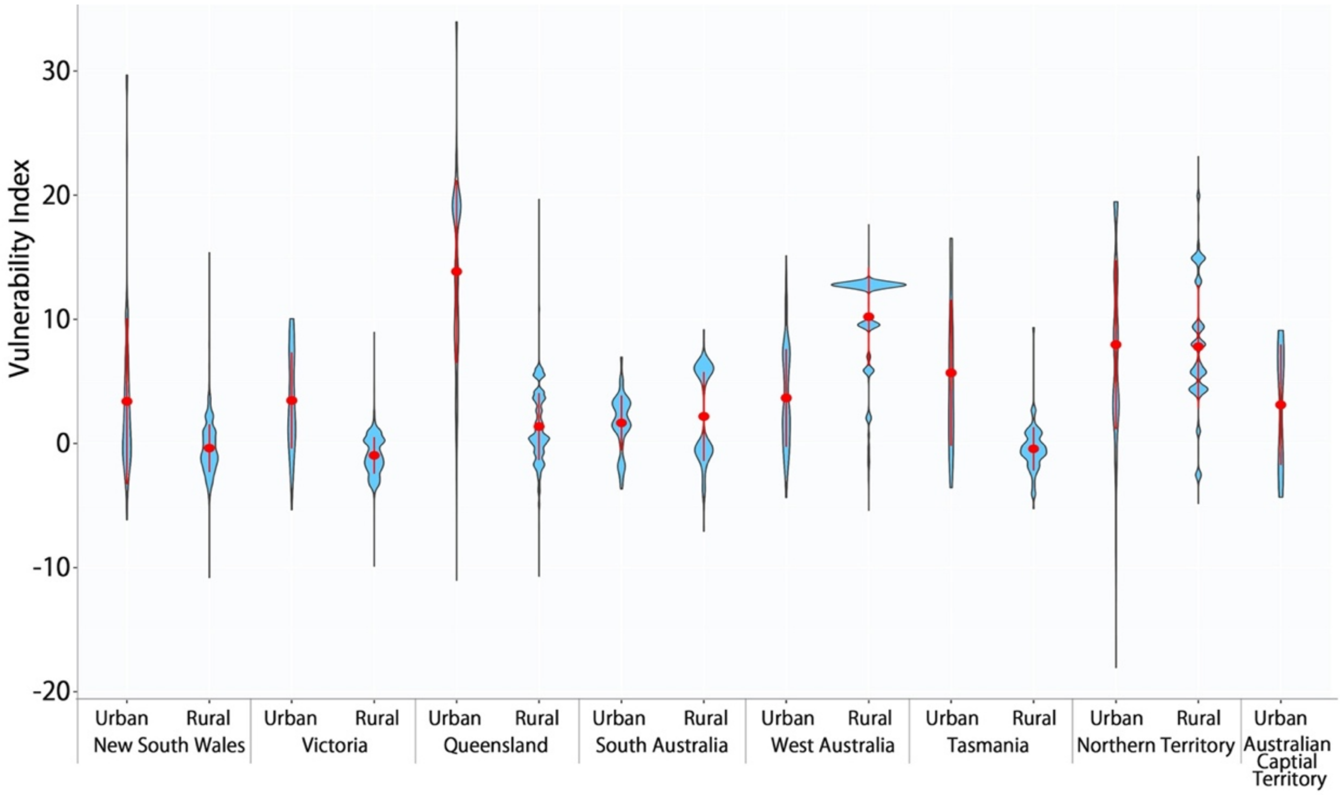

Figure S10. Distribution of the vulnerability index in wildfire-affected areas (statistical details in Supplementary Table S11)

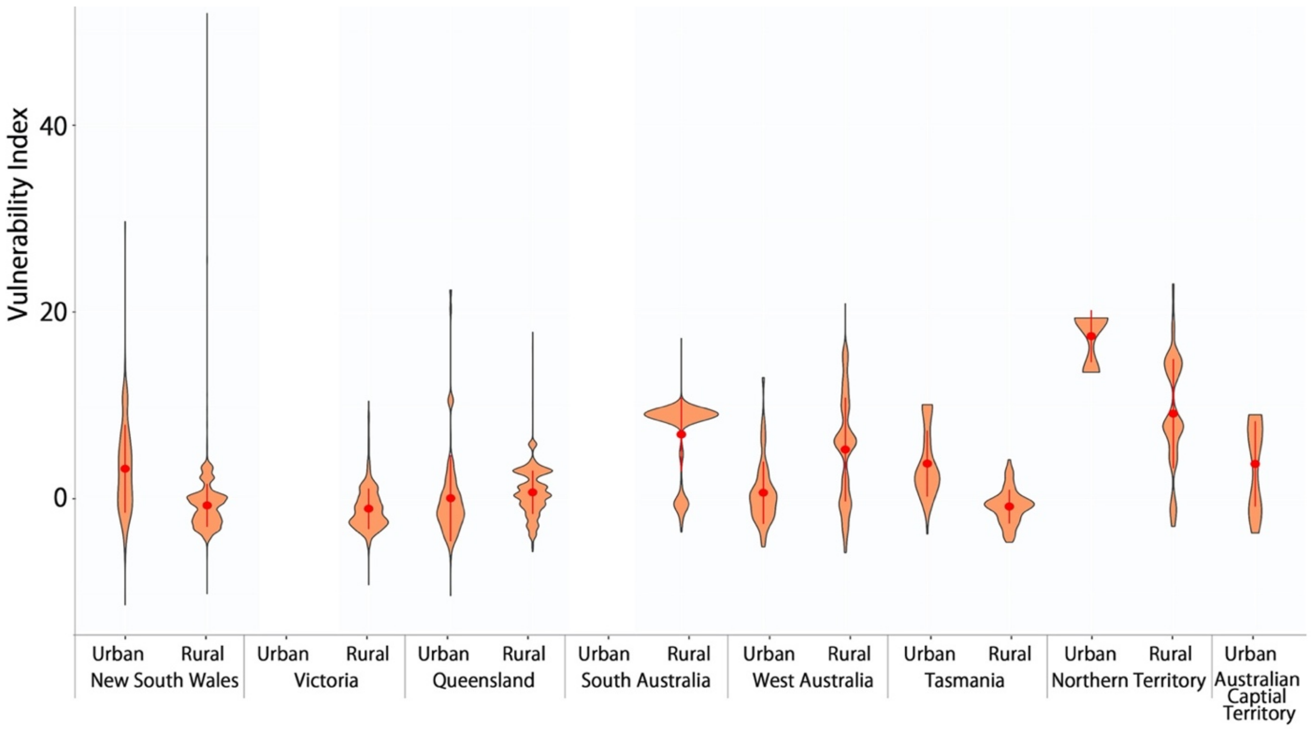

Figure S11. Distribution of the vulnerability index in flood-affected areas (statistical details in Supplementary Table S12)

Table S12. Statistic summary of vulnerability index in wildfire-affected and non-hazard areas

Region	Mean	Std. Dev	Min	Max	Number
Hazard-affected areas					
Sydney	3.39	6.70	-6.15	29.67	978
Rest of NSW	-0.38	1.94	-10.81	15.36	7769
Melbourne	3.46	3.88	-5.35	10.04	302
Rest of Vic.	-0.97	1.48	-9.89	8.95	5082
Brisbane	13.84	7.37	-11.02	33.96	6724
Rest of Qld	1.36	2.71	-10.70	19.66	242810
Adelaide	1.66	2.22	-3.67	6.94	190
Rest of SA	2.17	3.60	-7.08	9.16	3427
Perth	3.66	3.95	-4.35	15.11	473
Rest of WA	10.20	3.97	-5.40	17.62	64838
Hobart	5.68	5.90	-3.56	16.51	152
Rest of Tas.	-0.43	1.76	-5.25	9.32	1737
Darwin	7.96	6.82	-18.06	19.47	1730
Rest of NT	7.80	4.96	-4.84	23.10	311277
Australian Capital Territory	3.11	4.86	-4.33	9.10	44
Non-hazard areas					
Sydney	1.32	4.40	-14.04	33.24	11098
Rest of NSW	-0.08	2.78	-10.81	59.57	9908
Melbourne	-0.48	2.43	-15.26	51.24	10004
Rest of Vic.	-0.45	2.14	-9.89	17.12	5193
Brisbane	2.42	6.71	-20.27	33.96	6092
Rest of Qld	2.00	2.55	-10.70	69.09	136627
Adelaide	0.15	2.47	-30.42	13.00	3069

Rest of SA	1.73	3.66	-21.99	23.52	2616
Perth	-0.66	2.60	-16.37	15.11	4419
Rest of WA	9.01	5.41	-12.82	48.50	18553
Hobart	1.85	4.29	-11.49	16.51	660
Rest of Tas.	0.01	2.11	-5.25	13.22	1614
Darwin	5.40	7.17	-18.06	19.47	937
Rest of NT	8.29	4.80	-4.84	25.33	138582
Australian Capital Territory	-2.44	2.19	-19.22	11.57	1027

Table S13. Statistic summary of vulnerability index in flood-affected and non-hazard areas

**Region**	**Mean**	**Std. Dev**	**Min**	**Max**	**Number**
Hazard-affected areas					
Sydney	3.21	4.73	-6.15	29.67	978
Rest of NSW	-0.71	2.32	-10.81	15.36	7769
Melbourne	—	—	—	—	—
Rest of Vic.	-0.95	2.19	-9.89	8.95	5082
Brisbane	0.17	4.62	-11.02	33.96	6724
Rest of Qld	0.81	2.33	-10.70	19.66	242810
Adelaide	—	—	—	—	—
Rest of SA	7.00	3.97	-7.08	9.16	3427
Perth	0.77	3.35	-4.35	15.11	473
Rest of WA	5.38	5.58	-5.40	17.62	64838
Hobart	3.88	3.55	-3.56	16.51	152
Rest of Tas.	-0.71	1.83	-5.25	9.32	1737
Darwin	17.53	2.79	-18.06	19.47	1730
Rest of NT	9.23	5.86	-4.84	23.10	311277
Australian Capital Territory	3.85	4.59	-4.33	9.10	44
Non-hazard areas					
Sydney	0.90	3.02	-14.04	33.24	10759
Rest of NSW	-0.42	2.83	-10.81	59.57	11149
Melbourne	-0.49	2.41	-15.26	51.24	9982
Rest of Vic.	-0.58	2.29	-9.89	17.12	4092
Brisbane	0.21	3.21	-20.27	33.96	4968
Rest of Qld	0.29	2.76	-10.70	69.09	10042
Adelaide	0.12	2.46	-30.42	13.00	3039
Rest of SA	3.73	4.93	-21.99	23.52	2053
Perth	-0.86	2.30	-16.37	15.11	4319
Rest of WA	3.53	6.03	-12.82	48.50	3816
Hobart	0.93	3.48	-11.49	16.51	574
Rest of Tas.	0.19	2.42	-5.25	13.22	1020
Darwin	-0.47	4.25	-18.06	19.47	315
Rest of NT	10.18	5.83	-4.84	25.33	1129
Australian Capital Territory	-2.45	2.17	-19.22	11.57	1026

Table S14. Inequity index of vulnerability in hazard-affected areas

	New South Wales		Victoria		Queensland		South Australia		West Australia		Tasmania		Northern Territory		Australian Capital Territory
	**Urban**	**Rural**	**Urban**	**Rural**	**Urban**	**Rural**	**Urban**	**Rural**	**Urban**	**Rural**	**Urban**	**Rural**	**Urban**	**Rural**	**Urban**
Wildfire															
k=0.25	-1.031	-0.526	-1.225	-0.347	-2.174	-0.670	-0.758	-0.424	-1.079	-0.985	-1.786	-0.317	-4.829	-5.433	-1.019
k=0.5	-1.695	-1.086	-2.059	-0.740	-4.186	-1.269	-1.348	-0.801	-1.777	-1.463	-2.709	-0.606	-10.634	-7.927	-1.545
k=0.75	-2.234	-2.064	-2.665	-1.403	-6.650	-2.212	-1.777	-1.234	-2.303	-1.833	-3.307	-0.892	-13.462	-9.185	-1.881
Earthquake															
k=0.25	-0.969	-0.874	-0.639	-0.614	—	-4.742	-2.620	-0.706	-0.677	-1.153	—	-0.440	-2.950	-4.641	-0.795
k=0.5	-1.948	-1.616	-1.435	-1.174	—	-6.533	-14.519	-1.350	-2.330	-2.197	—	-0.779	-7.903	-6.928	-3.989
k=0.75	-5.740	-3.894	-5.174	-2.778	—	-11.007	-29.772	-3.376	-8.237	-6.198	—	-1.641	-16.091	-12.224	-11.658
Flood															
k=0.25	-1.774	-0.889	—	-0.753	-0.665	-0.647	—	-0.675	-0.807	-1.803	-0.873	-0.426	-0.969	-4.792	-2.240
k=0.5	-4.168	-1.634	—	-1.396	-1.269	-1.141	—	-1.093	-1.376	-2.664	-1.594	-0.798	-1.619	-6.985	-3.387
k=0.75	-6.650	-2.606	—	-2.124	-2.047	-1.576	—	-1.402	-1.832	-3.275	-2.306	-1.123	-1.991	-8.041	-4.005

Table S15. Reclassification of PoI data with data format indicated in the bracket

	**Main type (format)**
1	Building (area)
2	Railway (line)
3	Road (line)
4	Traffic connections (point): crossing, roundabout, motorway junctions, slipway, stop signs, traffic signals, and turning circles.
5	Parking (point): on-ground, underground, multistorey parking, bicycle parking, vending parking
6	Services (point): gas/fuel station
7	City/town centres (point)
8	Streep lamps (point)
9	Transport stops (point): stops of all types of public transit
10	Public transits (area): bus, tram, railway, ferry
11	Relax design (point): bench
12	Dining (point): café, bar, fast food, pub, nightclub, food court, biergarten, bed and breakfast, restaurant,
13	Commercial: Supermarket, convenience, hairdresser, clothes, pharmacy, beverages, bakery, butcher, car dealership, department store, beauty shops, laundry, bicycle shops, gift shops, newsagent, furniture shop, greengrocer, jeweller, florist, outdoor shop, bookshop, shoe shop, sports shop, chemist, mobile phone shop, cinema, optician, computer shops, toy shops, general, market place, mall, video shops.
14	Recreational (point): tourist Information centre, viewpoint, camp site, picnic site, attraction, memorial, monument, caravan site, part, fountain, swimming pool, observation tower, archaeological, car rental, bicycle rental, theatre, garden centre, lighthouse, dog park, golf course, theme park, zoo, castle, ice rink, battlefield, playground, sport centre, stadium,
15	Communication (point): telecommunication towers
16	Financial (point): bank, ATM
17	Art (point): artwork, museum, art centre.
18	Accommodation (point): hotel, hostel, motel, guesthouse
19	Healthcare facilities (point): hospital, Doctors, dentist, nursing home
20	Education (point): School, kindergarten, college, university,
21	Waster facilities (point): recycling, recycling clothes, wastewater plant, recycling paper, recycling metal,
22	Public services (point): town hall, embassy, public building, post box, telephone, post office, Police station, Fire station, community centre, library
23	Veterinary (point)
24	Drinking water (point)

Note 6 (Generating NDBI)

The below code is used to generate NDBI in the developer's portal via Google Earth Engine.

```
var visParam = {
  min:157.8104761904762,
  max:2002.4276190476191,
   bands: ['B4','B3','B2'],
}
var s2 = ee.ImageCollection("COPERNICUS/S2");
var filtered = s2.filter(ee.Filter.lt('CLOUDY_PIXEL_PERCENTAGE', 25))
  .filter(ee.Filter.date('2020-10-01', '2021-03-31'))
  .filter(ee.Filter.bounds(australia))

var image = filtered.median();
Map.addLayer(image, visParam, 'collection');

// Calculate Normalized Difference Vegetation Index (NDVI)
// 'SWIR' (B11) and 'NIR' (B8)
var ndvi = image.normalizedDifference(['B11', 'B8']).rename(['ndbi']);
var palette = ['blue','white','red']
var ndviVis = {min:-1, max:1, palette: palette}
Map.addLayer(ndvi.clip(australia), ndviVis, 'ndbi')

// mean land surface temperature value for SA1
var mean = ndvi.reduceRegions({
  collection: sa1,
reducer: ee.Reducer.mean(),
  scale: 10, //metres
});
// // // Exporting image as CSV and GEOTIFF
Export.table.toDrive({
collection: mean,
  folder: 'imageToDriveExample',
fileFormat: 'csv',
});
```

Table S16. Sensitivity analysis of vulnerability index in urban areas based on three scenarios

**Scenario 1 — 10 PC**		
Cumulative % = 69.45<br>KMO test = 0.794 (Sig < 0.001)<br>Bartlett's test of sphericity = 1299179		
PC 1	No car	0.799
	Multi-unit houses	0.779
	Renters	0.764
PC 2	Age above 65	0.942
	Median age	0.924
	Not labour force	0.806
	Need assistance	0.706
PC 3	Non-English speaker	0.909
	Minority	0.859
	Bad English	0.835
PC 4	Distance to commercial	0.841
	Distance to public transits	0.782
	Distance to public service	0.728
	Distance to healthcare	0.715
PC 5	Non-MPA occupation	0.859
	Low education	0.761
PC 6	Connectivity design	0.999

**Scenario 2 —12 PC**		
Cumulative % = 74.58<br>KMO test = 0.794 (Sig < 0.001)<br>Bartlett's test of sphericity = 1299179		
PC 1	Age above 65	0.938
	Median age	0.908
	Not labour force	0.789
	Need assistance	0.721
PC 2	Renters	0.835
	No car	0.803
	Multi-unit houses	0.798
PC 3	Non-English speaker	0.908
	Minority	0.858
	Bad English	0.837
	Ethnic diversity	0.714
PC 4	Non-MPA occupation	0.865
	Low education	0.768
	Low income	0.700
PC 5	Distance to commercial	0.842
	Distance to public transits	0.786
	Distance to public service	0.732

**Scenario 3 —17 PC**		
Cumulative % = 84.69<br>KMO test = 0.794 (Sig < 0.001)<br>Bartlett's test of sphericity = 1299179		
PC 1	Age above 65	0.931
	Median age	0.898
	Not labour force	0.819
	Need assistance	0.732
PC 2	Non-English speaker	0.905
	Minority	0.891
	Bad English	0.791
	Ethnic diversity	0.767
PC 3	Renters	0.846
	No car	0.804
	Multi-unit houses	0.766
PC 4	Non-MPA occupation	0.822
	Low income	0.798
	Low education	0.779
PC 5	Density of roads and railways	0.999
PC 6	Distance to commercial	0.830
	Distance to public service	0.801

	Density of buildings	0.999
	Density of roads and railways	0.999
PC 7	No internet	0.619
PC 8	Distance to cities	0.796
PC 9	Mobile living	-0.782
PC 10	Street design	0.886

	Distance to healthcare	0.717
PC 6	Connectivity design	0.999
	Density of buildings	0.999
	Density of roads and railways	0.999
PC 7	Need child care	0.668
	Age 0-18	0.636
PC 8	Retired village	0.846
PC 9	Distance to cities	0.835
PC 10	Mobile living	0.973
PC 11	Diversity of land use	0.971
PC 12	Street design	0.998

	Distance to healthcare	0.774
	Distance to public transits	0.733
PC 7	Need child care	0.743
	Age 0-18	0.703
	Not married	0.686
PC 8	Retired village	0.961
PC 9	Indigenous	0.867
PC 10	Distance to cities	0.972
PC 11	Female	0.908
PC 12	Mobile living	0.989
PC 13	Diversity of housing	0.939
PC 14	Multi-family HH	0.807
PC 15	Diversity of land use	0.990
PC 16	Street design	0.999
PC 17	Distance to roads	0.848

Table S17. Pairwise Pearson's correlation of vulnerability index in urban areas (N=55,195)

	**Scenario 1 (10 PC)**	**Scenario 2 (12 PC)**	**Scenario 3 (17 PC)**
Scenario 1 (10 PC)	1		
Scenario 2 (12 PC)	.813**	1	
Scenario 3 (17 PC)	.761**	.773**	1

Note: **: p<0.01; PC = principal components

Table 18. One sample T statistics of vulnerability index in urban areas

	**T-statistics**	**Sig. (2-tailed)**	**Mean Difference**	**95% CI**
Scenario 1 (10 PC)	0	1	0.00E+00	(-0.0331, 0.0331)
Scenario 2 (12 PC)	0	1	-1.43E-12	(-0.0362, 0.0362)
Scenario 3 (17 PC)	0	1	-2.36E-12	(-0.0431, 0.0431)

Note: CI = Confidence intervals

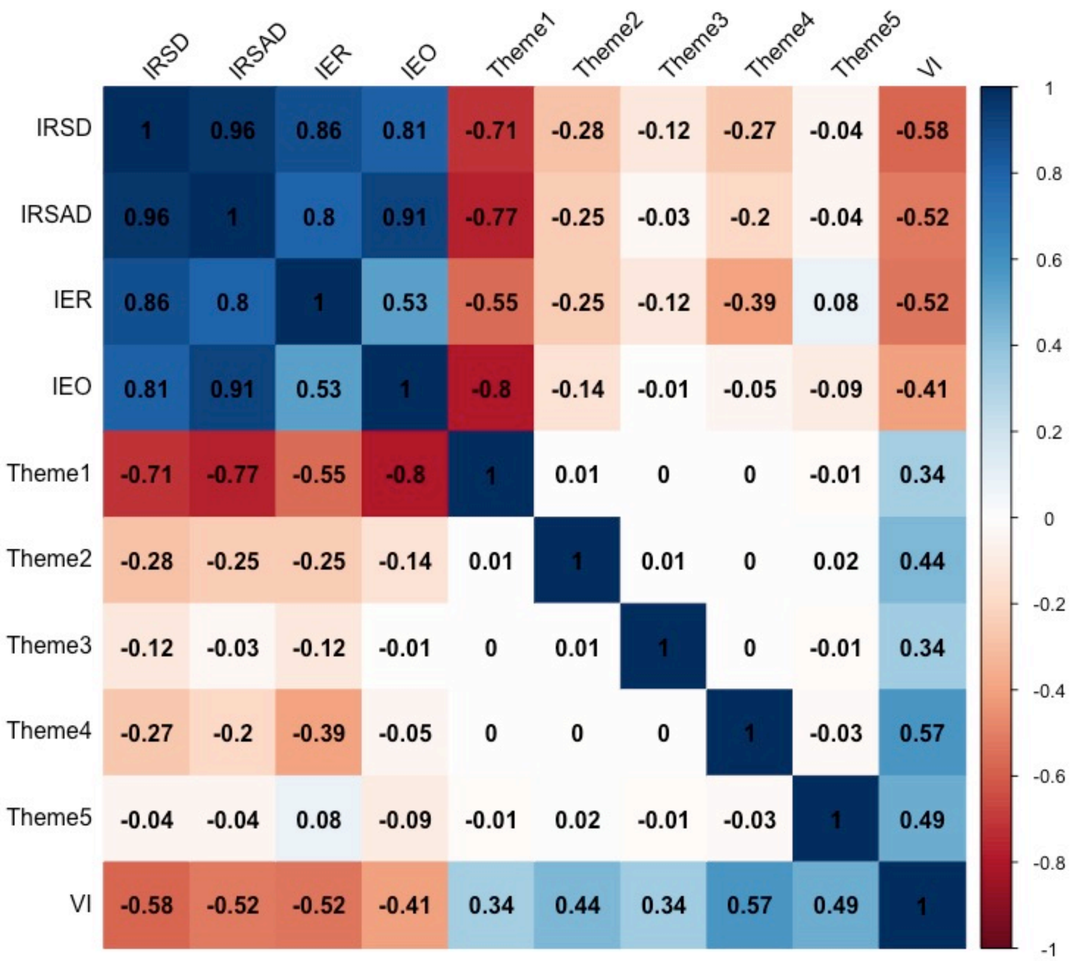

Figure S12. Correlations between four indices of SEIFA and our measures of vulnerability index. IRSD = Index of Relative Socio-Economic Disadvantage; IRSAD = Index of Relative Socio-Economic Advantage and Disadvantage; IEO = Index of Education and Occupation; IER = Index of Economic Resources; VI = vulnerability index; Theme 1: socioeconomic status; Theme 2: demographic composition & disability; Theme 3: minority status & language; Theme 4: housing type & transportation; Theme 5: built environment

Supplementary Reference

Australia Bureau of Statistics (2016). 1270.0.55.001 - Australian Statistical Geography Standard (ASGS): Volume 1 - Main Structure and Greater Capital City Statistical Areas, July 2016. Available at: https://www.abs.gov.au/ausstats/abs@.nsf/Lookup/by%20Subject/1270.0.55.001~July%202016~Main%20Features~Greater%20Capital%20City%20Statistical%20Areas%20(GCCSA)~10003

Australia Bureau of Statistics (2022). Population. Available at: https://www.abs.gov.au/statistics/people/population

Akinola, O. V., J. Adegoke and T. E. Ologunorisa (2019). "Assessment of Social Vulnerability to Wildfire in Missouri, United States of America." Journal of Sustainable Development **12**(4).

Aksha, S. K., L. Juran, L. M. Resler and Y. Zhang (2019). "An analysis of social vulnerability to natural hazards in Nepal using a modified social vulnerability index." International Journal of Disaster Risk Science **10**(1): 103-116.

Berke, P., S. Yu, M. Malecha and J. Cooper (2019). "Plans that disrupt development: Equity policies and social vulnerability in six coastal cities." Journal of Planning Education and Research: 0739456X19861144.

Brodie, M., E. Weltzien, D. Altman, R. J. Blendon and J. M. Benson (2006). "Experiences of Hurricane Katrina evacuees in Houston shelters: Implications for future planning." American Journal of Public Health **96**(8): 1402-1408.

Burton, C. and S. L. Cutter (2008). "Levee failures and social vulnerability in the Sacramento-San Joaquin Delta area, California." Natural hazards review **9**(3): 136-149.

Center, E. H. J. H. I. and H. H. John (2000). The hidden costs of coastal hazards: Implications for risk assessment and mitigation, Island Press.

Chen, W., S. L. Cutter, C. T. Emrich and P. Shi (2013). "Measuring social vulnerability to natural hazards in the Yangtze River Delta region, China." International Journal of Disaster Risk Science **4**(4): 169-181.

Cumberbatch, J., C. Drakes, T. Mackey, M. Nagdee, J. Wood, A. K. Degia and C. Hinds (2020). "Social Vulnerability Index: Barbados–A Case Study." Coastal Management **48**(5): 505-526.

Cutter, S. L., K. D. Ash and C. T. Emrich (2014). "The geographies of community disaster resilience." Global environmental change **29**: 65-77.

Cutter, S. L., B. J. Boruff and W. L. Shirley (2003). "Social vulnerability to environmental hazards." Social science quarterly **84**(2): 242-261.

Cutter, S. L., J. T. Mitchell and M. S. Scott (2000). "Revealing the vulnerability of people and places: A case study of Georgetown County, South Carolina." Annals of the association of American Geographers **90**(4): 713-737.

de Oliveira Mendes, J. M. (2009). "Social vulnerability indexes as planning tools: beyond the preparedness paradigm." Journal of Risk Research **12**(1): 43-58.

Drakes, O., E. Tate, J. Rainey and S. Brody (2021). "Social vulnerability and short-term disaster assistance in the United States." International Journal of Disaster Risk Reduction **53**: 102010.

Flanagan, B. E., E. W. Gregory, E. J. Hallisey, J. L. Heitgerd and B. Lewis (2011). "A social vulnerability index for disaster management." Journal of homeland security and emergency management **8**(1).

Holand, I. S., P. Lujala and J. K. Rød (2011). "Social vulnerability assessment for Norway: A quantitative approach." Norsk Geografisk Tidsskrift-Norwegian Journal of Geography **65**(1): 1-17.

Kirby, R. H., M. A. Reams, N. S. Lam, L. Zou, G. G. Dekker and D. Fundter (2019). "Assessing social vulnerability to flood hazards in the Dutch Province of Zeeland." International Journal of Disaster Risk Science **10**(2): 233-243.

Krishnan, P., P. S. Ananthan, R. Purvaja, J. J. J. Jeevamani, J. A. Infantina, C. S. Rao, A. Anand, R. S. Mahendra, I. Sekar and K. Kareemulla (2019). "Framework for mapping the drivers of coastal vulnerability and spatial decision making for climate-change adaptation: A case study from Maharashtra, India." Ambio **48**(2): 192-212.

Kumar, D. and R. K. Bhattacharjya (2020). "Study of Integrated Social Vulnerability Index SoVI of Hilly Region of Uttarakhand, India." Environmental and Climate Technologies **24**(1): 105-122.

Mainali, J. and N. G. Pricope (2017). "High-resolution spatial assessment of population vulnerability to climate change in Nepal." Applied Geography **82**: 66-82.

Martin, S. D., A. C. Bush and J. A. Lynch (2006). "A national survey of terrorism preparedness training among pediatric, family practice, and emergency medicine programs." Pediatrics **118**(3): e620-e626.

McGuire, L. C., E. S. Ford and C. A. Okoro (2007). "Natural disasters and older US adults with disabilities: Implications for evacuation." Disasters **31**(1): 49-56.

Mileti, D. (1999). Disasters by design: A reassessment of natural hazards in the United States, Joseph Henry Press.

Morrow, B. H. (1999). "Identifying and mapping community vulnerability." Disasters **23**(1): 1-18.

Nicholson, A., A. Attal-Juncqua and S. Wollek (2021). "Exploring Disaster Human Services for Children and Youth."

Peek-Asa, C., M. Ramirez, H. Seligson and K. Shoaf (2003). "Seismic, structural, and individual factors associated with earthquake related injury." Injury prevention **9**(1): 62-66.

Prosdocimi, D. and K. Klima (2020). "Health effects of heat vulnerability in Rio de Janeiro: a validation model for policy applications." SN Applied Sciences **2**(12): 1-11.

Rosenkoetter, M. M., E. K. Covan, B. K. Cobb, S. Bunting and M. Weinrich (2007). "Perceptions of older adults regarding evacuation in the event of a natural disaster." Public Health Nursing **24**(2): 160-168.

Schmidtlein, M. C., R. C. Deutsch, W. W. Piegorsch and S. L. Cutter (2008). "A sensitivity analysis of the social vulnerability index." Risk Analysis: An International Journal **28**(4): 1099-1114.

Solangaarachchi, D., A. L. Griffin and M. D. Doherty (2012). "Social vulnerability in the context of bushfire risk at the urban-bush interface in Sydney: a case study of the Blue Mountains and Ku-ring-gai local council areas." Natural hazards **64**(2): 1873-1898.

Tierney, K. (2011). Social inequality, hazards, and disasters. On risk and disaster, University of Pennsylvania Press: 109-128.

Wigtil, G., R. B. Hammer, J. D. Kline, M. H. Mockrin, S. I. Stewart, D. Roper and V. C. Radeloff (2016). "Places where wildfire potential and social vulnerability coincide in the coterminous United States." International Journal of Wildland Fire **25**(8): 896-908.
